# Alternatives to animal models in gastroenterology and hepatology research

**DOI:** 10.3389/fphar.2026.1789616

**Published:** 2026-03-27

**Authors:** Elena Gardey, Anja R. Geisler, Alina Löser, Andreas Stallmach, Anna P. Kipp, Stefan Lorkowski, Maria Witt-Wallert

**Affiliations:** 1 Department of Internal Medicine IV (Gastroenterology, Hepatology, Infectious Diseases and Interdisciplinary Endoscopy), Jena University Hospital, Jena, Germany; 2 Department of Nutritional Biochemistry and Physiology, Institute of Nutritional Science, Friedrich Schiller University Jena, Jena, Germany; 3 Department of Nutritional Physiology, Institute of Nutritional Science, Friedrich Schiller University Jena, Jena, Germany

**Keywords:** everted sac, *ex vivo* model, *in vitro* model, organoids, organ-on-chip, precision cut tissue slices, spheroids, Ussing chamber

## Abstract

The accurate definition of *in vivo*, *ex vivo* and *in vitro* models is critical for the whole R&D process, i.e., basic research, clinical translation and reliability of results. Although many models are currently being developed, it is important to recognize the limitations and advantages of each of them. The aim of this review is to compile the most important alternatives to animal models in the fields of gastroenterology and hepatology research. A thorough comparison and understanding of each alternative model will certainly save time and money and will lead to better predictability and reliability of results for clinical translation and clinical trials. There is no single model capable of replacing the complexity of human biology. Nevertheless, it is essential to understand the fundamentals of human anatomy, physiology and disease pathophysiology to select the most appropriate model in translational research. At the same time, the appropriate model must be selected to gain a deeper understanding of the principal processes underlying human physiology and the pathology of diseases.

## 1 Introduction

An animal model is an important tool for basic research as well as translational research in the fields of hepatology and gastroenterology. In addition to its extensive use in scientific research, the animal model is also a crucial instrument for preclinical studies, pharmacological studies, and nutritional research. It is important to note, however, that some animal models may not be entirely applicable to clinical settings due to discrepancies in the structure of the organs, e.g., liver and gastrointestinal (GI) tract. In general, the liver architecture of rodents and humans has more similarities than differences, whereby the differences are mainly quantitative in nature. The biggest difference concerns the macroscopic lobar structure, as rodents have a clearly lobulated liver, whereas the human liver is essentially non-lobulated. The main differences in microscopic lobular architecture is the amount of connective tissue in the portal tracts and the dependence of perfusion on the hepatic artery in larger mammals (Kruepunga et al., 2019). While adverse effects related to cardiovascular, gastrointestinal, and hematologic toxicity can be predicted with an accuracy of 85%–90% in animal studies, the predictive value for hepatotoxicity in such models is limited to approximately 50% (Olson et al., 2000; Ware and Khetani, 2017).

For the GI tract there are numerous physiological differences between human and animal physiology, including the thickness of the mucus layer, the number of microfold (M) cells, the absence of a gallbladder in rats, the lack of an emetic reflex, and the pronounced cecum (Johnson and Greenwood-Van Meerveld, 2017). It was demonstrated that rats are unable to reproduce the metabolic processes and drug bioavailability due to alterations in the underlying molecular mechanisms (Jung and Kim, 2021; Cao et al., 2006). Furthermore, it is challenging to investigate the microbiome using an animal model, as some pathogens, such as *Listeria monocytogenes*, are unable to infect rodents (Drolia et al., 2018). Moreover, there are pronounced differences between the pH ranges, transit times, and intestinal lengths and permeability values observed in humans and animals.

The permeability values of mice are comparable to those observed in humans but are different in rats and pigs. Data from dogs (e.g., Beagle) remain limited. The transit time for the fasted colon is 8–18 h for humans and up to 3 days for pigs. The pH range in the fasted stomach is 1.0–3.5 for humans, 4.0 for mice, 4.5–7.0 for rats, 1.5–1.8 for dogs and 1.2–4.0 for pigs (Xu et al., 2021; Sjögren et al., 2014; Lui et al., 1986; Kararli, 1995). As demonstrated, pig models exhibit a higher degree of physiological similarity with the human GI tract, resulting in data with higher relevance for humans. However, the dimensions of these animals and the requirements for their welfare are not compatible with the facilities typically available in standard laboratories (Lunney et al., 2021). A further challenge that must be overcome, especially in the field of nutritional research, is the fact that rodents, dogs and pigs have different dietary requirements. Therefore, due to the differences in physiology between animals and humans, it is important to interpret the results and conclusions of animal experiments with caution.

The establishment of reliable disease models in animals presents a significant challenge (Loewa et al., 2023; Özkan et al., 2024). Disease conditions such as inflammatory bowel disease (IBD) and steatotic liver disease (SLD), like metabolic dysfunction-associated steatotic liver disease (MASLD), exhibit considerable heterogeneity among patients. They are characterized by complex pathogenesis, complex multifactorial etiologies, species-specific differences as well as lack of comorbidities such as pattern recognition receptor-associated gene polymorphisms, allergies, microbiome and lipid metabolism imbalances. These factors contribute to the difficulties in developing a representative and exemplary IBD model and a model for MASLD (Ho et al., 2019).

The use of animal models in research is primarily constrained by ethical considerations, as all animal experiments must be approved by an ethical committee and justified in terms of potential benefit versus animal welfare concerns. In addition, financial and time-related costs, as well as the limited flexibility to rapidly adjust experimental conditions, further restrict their use. Consequently, there is a clear need to develop alternative models in hepatology and gastroenterology that minimize or replace animal use (Jung and Kim, 2021). These models could be used to study pathogenesis of disease, drug development, toxicity studies, and other relevant areas of research. Currently, there are numerous *in vitro* and *ex vivo* alternative models with varying degrees of reliability that can be useful in this regard. A number of available alternative models help to minimize the number of animal experiments in accordance with the 3Rs (Replace, Reduce, Refine) concept. These include *in vitro* cell culture models, organ-on-chip models, 3D organoids and *ex vivo* experiments, including the Ussing chamber technique, the everted sac method and precision-cut tissue slices (PCTS). Although *in vitro* 2D cell culture models are the most commonly used systems, there are some relevant limitations. For example, immortalized cells exhibit a distinct genetic profile compared to primary cells. While there are clear benefits to the long-term cultivation of cell lines, this can potentially result in mutations and alterations to their characteristics (Weiskirchen, 2022). Furthermore, erroneous identification of cell lines, cross-contamination, and *Mycoplasma* contamination are frequently observed (Arzumanian et al., 2021). *Ex vivo* experiments represent an effective means of utilizing animal organs in a manner that exemplifies the principles of the *Reduce* and *Refine* concept as part of the 3R. In *ex vivo* experiments, the *Replace* concept can be implemented using human organs or biopsies (Othman et al., 2020).

This review will address the potential of alternatives to animal models in the fields of hepatology and gastroenterology research, with a particular focus on their advantages and limitations. Furthermore, this paper will address the concepts and approaches involved in selecting an alternative model.

## 2 Cellular 2D models

Two-dimensional (2D) cell culture systems are widely used in gastroenterology and hepatology research because they are a cost-effective, technically simple, and highly reproducible approach for *in vitro* experiments studying basic cellular processes under controlled conditions. They facilitate high-throughput screening and are widely used for preliminary pharmacological and toxicological evaluations. However, 2D cultures predominantly rely on monocultures, thereby lacking heterotypic cell–cell interactions and crosstalk between the epithelial, immune, stromal and endothelial cells that are critical for tissue function *in vivo*. In the liver, this limits the modeling of inflammatory responses and metabolic zonation, while in the gut, it impairs the representation of epithelial barrier dynamics and host–microbiome interactions. Additionally, 2D cultures are typically grown on rigid plastic surfaces. Although coating materials (e.g., gelatin or collagen) can enhance attachment, they do not replicate the composition or mechanics of the native extracellular matrix (ECM), such as the soft, porous structure of the liver sinusoid or the stratified basement membrane of the intestinal mucosa.

Consequently, 2D cultures fail to accurately mimic the complex three-dimensional architecture, extracellular matrix interactions, and microenvironmental gradients found *in vivo*. As a result, they often exhibit altered cell morphology, gene expression, and functional responses, limiting their translational relevance for disease modeling and therapeutic testing (Borah and Kumar, 2022; Cardoso et al., 2023).

### 2.1 Gastroenterology

Cancer coli-2 (Caco-2) cells are a gold standard cell line in gastroenterology. These cells were derived from human colon adenocarcinoma and are capable of forming tight junctions (TJ), apical microvilli, and expressing brush border enzymes (e.g., peptidase, alkaline phosphatase) and various transporters (e.g., P-gp, MRP, OTC1) (Xu et al., 2021; Kus et al., 2023; Ding et al., 2021). Epithelial cells cultured on a semipermeable membrane exhibit apical-basal polarity, brush border formation, and the presence of TJ between cells. The membrane is typically manufactured from polycarbonate, polyester, or polyethylene terephthalate. Complete differentiation of Caco-2 cells to mature enterocyte-like cells requires approximately 21 days (Kus et al., 2023; Ding et al., 2021; Natoli et al., 2012). To establish a differentiated Caco-2 model, a passage of 20–60 is typically required, with a cell density of 2.5 × 10^5^ cells/cm^2^ (Ding et al., 2021). The epithelial cells that are cultured on membrane inserts, i.e., Transwell® systems (see Chapter 2.3), form a semi-permeable physical barrier that exhibits increased transepithelial electrical resistance (TEER) over time (Martini et al., 2017; Srinivasan et al., 2015). The integrity of a cell monolayer can be investigated by quantifying the passage of fluorescein or a radiolabeled marker, such as ^3^H-mannitol (Biganzoli et al., 1999). Caco-2 cells can also be cultured on the surface of a subepithelial-like tissue matrix formed from intestinal or dermal fibroblasts (Darling et al., 2020).

To a certain extent, the cellular model represents a relatively simple epithelial barrier with certain properties that are similar to those observed *in vivo* for the real epithelial barrier. The cellular model is an effective method for investigating interactions between components present in the intestinal lumen and the host, which both are separated from each other by the epithelial layer and the integrity of the epithelial barrier (Grosheva et al., 2020; Viswanathan et al., 2008). This model is the most convenient for the study of drug absorption, distribution, metabolism, and excretion (ADME) studies (Kus et al., 2023; Fedi et al., 2021). Moreover, the Caco-2 cell model is recognized by Food and Drug Administration (FDA) and the European Medicines Agency (EMA) as a reliable model for predicting drug bioavailability (Kus et al., 2023). Regarding FDA and EMA, internal standardization and validation of the Caco-2 cell line is required. A validated cellular model must demonstrate a correlation between the apparent permeability coefficient (P_app_) and the *in vivo* human intestinal absorption of a minimum of five model drugs (Kus et al., 2023). The development and improvement of existing *in vitro* cellular models in the field of gastroenterology permit the use of more accurate tools for the study of drug permeability (Darling et al., 2020). Thus, human small intestinal epithelial cells (HIEC) can be an alternative to Caco-2 monolayers. HIEC differentiate from adult intestinal stem cells and can be used for more accurate prediction of oral drug absorption in humans (Takenaka et al., 2016). In addition, LS-174T and HT-29 cells, which exhibit goblet-like cell properties, or transplantable human carcinoma cell line (T-84) can be used. As an alternative, a non-carcinogenic intestinal porcine enterocyte cell line (IPEC-J2) exhibited a satisfactory degree of relevance to *in vivo* human physiology (Table 1) (Zweibaum et al., 1991; Devriese et al., 2017; Vergauwen, 2015).

**TABLE 1:** Most relevant cell lines in gastroenterology research.

Cell line/Cells	Origin	Advantages	Disadvantages
Caco-2	Human (colon adenocarcinoma)	Characteristics - Well-characterized, widely used, gold standard - FDA-approved Application - Prediction of oral drug absorption	- Deficient in native enzymes - Limited in predicting first-pass metabolism - Low endocytic activity - Thin glycocalyx layer - Tumor origin (genomic instability alters transporter and barrier properties)
LS-174T	Human (colon adenocarcinoma)	Characteristics - Production of mucin - Resemblance to goblet cells Application - Colorectal cancer research	- Tumor origin (genomic instability, mutations, and altered signaling pathways reduce physiological relevance)
MDCK	Madin-Darby (canine kidney)	Characteristics - Brush border and tight junctions - 3–5 days for full confluency Application - Drug permeability - Host-pathogen interactions	- Non-intestinal origin - Low homogeneity - Lack of transporters and metabolic enzymes
IPEC-J2	Porcine (intestinal enterocytes from jejunum of neonatal piglet)	Characteristics - Mimic human physiology Application - Epithelial transport - Host-pathogen interactions	- No mucus layer
Primary human intestinal epithelial cells	Human intestinal biopsies	Characteristics - Isolated from patients, ensuring clinical relevance Application - Human physiology	- Limited availability and ethical concerns - Variability between donors
T-84	Human (lung metastasis of colon carcinoma)	Characteristics - Form tight junctions - Polarized epithelial monolayers Application - Drug bioavailability - Host-pathogen interactions - Absorption, metabolism, drug bioavailability, epithelial barrier integrity	- Tumor origin (mutations and genomic instability can alter transporter expression and barrier physiology)
HT-29	Human (colon adenocarcinoma)	Characteristics - Similarities with enterocytes of the small intestine - Production of mucin Application - Drug transport - Molecular mechanisms of intestinal cell differentiation	- Low enzymatic activity - No expression of lactase - Tumor origin (genomic instability and mutations can reduce physiological relevance)

All models have their specific limitations and advantages. The Caco-2 cells model is an easy and cheap model for investigating drug absorption. The principal advantages of this simple model are its reproducibility, standardization, and capacity for high-throughput analyses. However, discrepancies between primary human epithelial cells and Caco-2 cells frequently lead to contradictory and inadequate results when compared to *in vivo* experiments. Caco-2 cells are deficient in native enzymes, show low expression of sodium glucose transporter 1, exhibit low endocytosis activity, and possess a markedly thin glycocalyx layer. Furthermore, the investigation of the commensal microbiome is not feasible in this *in vitro* model (Lundquist and Artursson, 2016; Jeong et al., 2025; Steffansen et al., 2017). In contrast, Madin-Darby canine kidney (MDCK) and IPEC-J2 cells are more suitable to investigate host-microbiome interactions. To select more suitable cellular models, it is essential to understand the limitations of each cell line which are outlined in Table 1. An appropriately selected cellular model can save time and money while enhancing the precision and biological pertinence of the results.

In conclusion, the cellular model is not capable of representing the complicated intestinal structure and is unsuitable for addressing more complex scientific questions (Fedi et al., 2021; Beterams et al., 2021). Nevertheless, the affordability and accessibility of the model, coupled with its simplicity, contribute to its widespread use and suitability as a first-choice assay. Furthermore, the investigation of certain scientific inquiries demands the application of the most fundamental model.

### 2.2 Hepatology

Human-derived hepatoblastoma (e.g., HepG2 (López-Terrada et al., 2009)) and hepatoma cell lines (e.g., Huh7), and primary hepatocytes, are among the most relevant models for liver research, particularly when studying human-specific liver functions and diseases. The epithelial-like HepG2 cells are of particular interest due to their extensive utilization and well-defined characteristics (Arzumanian et al., 2021). Derived from a human hepatoblastoma, these cells are a well-established model for the study of drug metabolism, liver toxicity, and cancer-related liver research (Arzumanian et al., 2021). However, hepatocytes undergo a metabolic shift, known as Warburg effect, when they transform into tumor cells (Perrin-Cocon et al., 2021). The energy metabolism and the metabolic gene profile of cancer cell lines, such as HepG2 and Huh7, do not adequately represent the metabolic gene and protein expression profiles of primary human hepatoma cells or liver tissues, respectively (Schicht et al., 2022). Therefore, HepG2 cells do not perfectly represent normal liver physiology due to their cancerous origin (Arzumanian et al., 2021). For example, they cannot secrete albumin. Nevertheless, they remain a valuable tool for investigating various liver functions, including protein synthesis and enzyme activity. However, to ascertain the applicability of the HepG2 cell line as a model of normal and pathological cellular processes, a case-by-case evaluation is required (Arzumanian et al., 2021). Thus, analysis of drug metabolism lacks validity, since proteins responsible for elimination of xenobiotics, the members of the cytochrome P450 family (CYP), are poorly expressed in HepG2 cells. Low or absent expression of CYP enzymes, including CYP2A6, CYP2C9, CYP2C19, CYP2D6, and CYP3A4, has been observed, thereby limiting their utility in studying phase I drug metabolism (Donato et al., 2015). However, it has been demonstrated that the metabolism of anticancer drugs and CYP inducers is permissible (Arzumanian et al., 2021; Takemura et al., 2021). Compared to liver tissue or primary hepatocytes, the characteristic hepatic transporters, sodium-taurocholate co-transporting polypeptide, bile salt export pump, and organic anion transporting polypeptide C are absent or poorly expressed in HepG2 cells (Guo et al., 2011; Hilgendorf et al., 2007).

In contrast, animal-derived cell lines, such as McA-RH7777 (rat) (American Type Culture Collection ATCC, 2025) and AML-12 (mouse) (ATTC, 2025), offer easier maintenance but may have limited relevance to human liver biology due to interspecies differences. McA-RH7777 is mainly used for rat liver cancer modeling and therapy research, while AML-12 is primarily used for studies of normal mouse liver cell biology, metabolism, and toxicology.

Primary human hepatocytes are still the gold standard since they represent the closest model to native human liver biology. They are particularly valuable for drug metabolism, toxicology testing, and disease modeling. However, their practical use is often hindered by challenges in maintaining cultures, donor variability, ethic approval, and their relatively short lifespan *in vitro* (Ruoß et al., 2020).

A promising alternative are HepaRG cells, which combine features of normal hepatocytes and liver progenitor cells. This makes them highly suitable for hepatology research, especially in areas like drug metabolism, liver disease modeling, and toxicology testing. They offer a more human-like model than many other cell lines, although their differentiation process and slower proliferation can be limiting factors in certain studies. Moreover, it is important to note that the utilization of HepaRG cells is subject to constraints imposed by proprietary licensing agreements. These licensing agreements typically necessitate the acquisition of a material transfer agreement (MTA), impose restrictions on the redistribution of materials, limit applications to non-commercial research unless additional permissions are obtained, and may incur usage-dependent fees. Despite these challenges, HepaRG cells are increasingly recognized for their relevance in research that requires a closer representation of human liver function (Jennen et al., 2010).

In summary, while HepG2 cells remain one of the most widely used and versatile models in hepatology research other human-derived cell lines like HepaRG and primary hepatocytes also play key roles, each with their own strengths and limitations (Table 2). These cell lines continue to advance our understanding of liver biology, disease, and pharmacology, but ongoing refinement and innovation in cell culture models promise to offer even more relevant human-based systems in the future.

**TABLE 2:** Most relevant cell lines in hepatology research.

Cell lines/cells	Origin	Advantages	Disadvantages
HepG2	Human (Hepatoblastoma)	Characteristics - Well-characterized - Widely used for liver-related research - Commercially available Application - Drug metabolism and liver toxicity studies	- Tumor origin (genomic instability and mutations can reduce physiological relevance) - Some phenotypic changes may alter results in certain assays
Huh7	Human (hepatocellular carcinoma)	Characteristics - Similar to HepG2 - More closely resemble PHH - Easy maintenance Application - Viral hepatitis studies, e.g., hepatitis-C-virus	- Tumor origin (genomic instability and mutations can reduce physiological relevance) - Possible genomic instability
Primary human hepatocytes (PHH)	Human (liver biopsies)	Characteristics - Mimicking human liver physiology - Isolated from patients, ensuring clinical relevance Application - Drug metabolism and toxicity testing	- Limited availability and ethical concerns - Difficult maintenance - Short lifespan (5 days vs. 3 weeks with collagen) (Gómez-Lechón et al., 1998) - Donor-specific variability
McA-RH7777	Rat (hepatoma)	Characteristics - Simple maintenance Application - Liver function studies - Investigation of liver injury and regeneration	- Species differences (rat vs. human) may limit direct relevance to human biology - Tumor origin, may not fully represent normal liver physiology
AML-12	Mouse (healthy hepatocyte)	Characteristics - Simple maintenance Application - *In vivo*-like studies in a controlled *in vitro* setting	- Species differences (murine vs. human) may limit direct relevance to human biology - Requires careful handling due to variability in mouse strain responses
HepaRG	Human (hepatoma-derived liver progenitor cells)	Characteristics - Closely resembles PHH - Proliferating HepaRG cells differentiate toward hepatocyte-like and biliary-like cells (Cerec et al., 2007) Application - Drug metabolism, toxicity, liver disease studies - Stable long-term culture (4 weeks) (Anthérieu et al., 2012; Klein et al., 2014)	- Differentiation for full hepatocyte-like function required - Not as fast-growing as some cancer-derived cell lines - Variability in differentiation efficiency

### 2.3 Co-culture models

The findings derived from monocultural cell lines are challenging to extrapolate to *in vivo* contexts, as organs such as the liver and intestine comprise diverse cell types with distinct functions. Co-culturing provides the opportunity to achieve more advanced models compared with single cell lines. Moreover, cultivation of different cells together provides an important tool to investigate the cell interaction but also produces challenges related to differences in growth rate and the ratio of different cell lines within the co-culture (Goers et al., 2014).

#### 2.3.1 Gastroenterology

**FIGURE 1:**
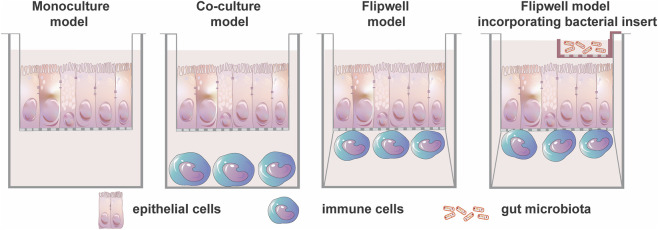
Schematic representation of mono-, co-culture and Flipwell models.

There are five main types of differentiated cells in the intestinal epithelium: enterocytes, goblet cells (mucus secreting cells), neuroendocrine cells, Paneth cells and M cells in the Peyer´s patches (Liévin-Le Moal and Servin, 2013). Thus, to create more complex and physiologically realistic models, co-culturing can be implemented. The co-culturing of different cell lines at once can be an alternative to monoculture cellular model, for example, Caco-2/HT29-MTX co-cultures and even triple Caco-2/HT29-MTX/Raji B co-cultures. In contrast to monocultures, higher physiological relevance can be achieved by adding mucus-producing HT29-MTX cells to Caco-2 cells. Moreover, Caco-2 incubated with Raji B lymphocytes have the potential to develop an M cell phenotype (Beterams et al., 2021; Lai and D’Souza, 2008; Lozoya-Agullo et al., 2017). These cellular models are widely used in drug research ranging from drug discovery to screening and bioavailability testing. Also in nutritional research, these models are used for e.g., nutraceutical testing, or absorption studies of natural components and nutrients. They are also employed in the study of transmembrane proteins (Fedi et al., 2021; Lozoya-Agullo et al., 2017; Rodrigues and Failla, 2021; Betge et al., 2022; Lancaster and Knoblich, 2014; Nunes et al., 2016). Furthermore, membrane inserts can be used to cultivate immune cells at the bottom of the well or beneath the insert, although this co-cultures may lack crucial cell-cell contacts that appear *in vivo*. Meanwhile, epithelial cells can be grown on the insert itself. Thus, a co-culture model represents a more complex system than the single cellular model (Figure 1) (Beamer et al., 2023). For instance, Beamer and colleagues recently demonstrated the novel 3D Flipwell model of the gut mucosal microenvironment for investigating interactions between the gut microbiota, epithelial cells, and immune cells. This model utilizes bottom-to-bottom inserts of a single layer of 0.4 µm pore size polyethylene terephthalate membrane (Lucchetti et al., 2024). A co-culture of Caco-2 and HT-29/MTX (9:1) cells are seeded on one side of the membrane, while THP-1 monocytes are seeded on the other side of the membrane. An additional insert containing bacteria could be placed on the epithelial insert. The novel model offers a distinct advantage over the model demonstrated by Noel et al. as it allows for the synchronized polarization of epithelial cells and differentiation of immune cells (Beamer et al., 2023; Noel et al., 2017).

#### 2.3.2 Hepatology

Up to 80% of cells within the liver are hepatocytes (Blouin et al., 1977), with interspecies differences (Baratta et al., 2009). However, to mimic the physiological environment and maintain hepatic functions, co-cultures with non-parenchymal cells (NPCs) are required (Table 3) (Arzumanian et al., 2021). Depending on the research focus, co-cultures can be established with macrophages, endothelial cells, and stellate cells. The polarization of macrophages to M1 and M2 further demonstrates differences in drug-induced liver injury (Kawase et al., 2022). Compared to the classical monocyte culture, interactions between liver cells can be mimicked, e.g., through the release of cytokines and metabolites (Krause et al., 2009). The advanced *Tissue Vault 2D Plus* (TV2D+) system is designed for the long-term cultivation of primary human hepatocytes (PHHs) by combining them with feeder cells, such as NPCs. PHHs are seeded on a collagen-coated plate together with feeder cells (NPCs) to provide structural support and cell signaling, and to promote the differentiation and survival of the PHHs by enabling the improved expression of liver-specific genes and enzymes (e.g., members of the CYP450 family), the secretion of growth factors, and cell–cell contact. Furthermore, this system offers stable metabolic activity and detoxification capabilities, enabling more accurate *in vivo* liver function modeling for drug testing, pharmacokinetic studies, and disease modeling (Odanga et al., 2023).

**TABLE 3:** Common co-cultures of hepatocytes, non-parenchymal cells (NPCs) and other cells.

​	Co- cells (NPCs)	Examples for cell type or cell line	Scope
Cell-cell system (direct)			
Primary human or murine hepatocytes	+ Macrophages	Kupffer cells	Investigation of immune response and inflammatory processes and LPS-mediated hepatotoxicity (Adams et al., 2010; Rose et al., 2016)
	+ Endothelial cells	Liver sinusoidal endothelial cells (LSECs), non-liver endothelial cells (e.g., human umbilical vein endothelial cells, HUVEC)	Modelling of drug exchange and vascular components (Clement et al., 1984)
	+ Stellate cells	LX-1/2	Mechanisms of fibrosis (Higashi et al., 2017) and storage of vitamin A (Senoo et al., 2007)
	+ Feeder cells (fibroblasts etc.), *Tissue Vault 2D Plus* (TV2D+) system	3T3-J2 fibroblasts	Long-term cultivation of PHH with extended hepatic functionality. Creating of a more physiologically relevant microenvironment (Odanga et al., 2023)
Human and murine hepatic cell lines (HepG2, AML12 etc.)	+ Macrophages	THP-1, RAW264.7, J774A.1	Studying liver-immune interactions, drug-induced liver injury, inflammation and metabolic responses (Kawase et al., 2022; Granitzny et al., 2017)
	+ Enterocytes	Caco-2	Studying gut–liver axis (Meroni et al., 2022)
Cell-supernatant system (indirect)			
Hepatocytes (primary or cell line)	+ Soluble factors (supernatant from immune cells)	​	Conclusion regarding the individual components in the supernatant
	+ Macrophages in Transwell systems	THP-1, RAW264.7, J774A.1	Communication via soluble compounds without direct cell–cell contact (Kawase et al., 2022)

Isolating the different primary liver cells is challenging. Therefore, model systems such as cancer cell lines (immortalized cell lines) are used. A common model is the combination of HepG2 and THP-1 cells in either direct or indirect co-culture.

Direct co-culture involves plating both cell types together on the same surface, enabling direct cell–cell interactions. However, this is less common due to the cells’ different adherence properties. Under physiological conditions, macrophages and hepatocytes occur at a ratio of 1:10 in the liver. The exact ratio needed for experiments depends on the desired intensity of the immune response, the differentiation of the THP-1 cells, the type of co-culture (direct or indirect) and the planned analysis. Thus, ratios of 1:0.4, 1:1 (Krause et al., 2009) and 1:2 were also used in practice (Boran et al., 2024). Co-cultures are maintained for 24–72 h, depending on the experimental endpoint. THP-1 cells were differentiated into macrophage-like cells using, e.g., phorbol 12-myristate 13-acetate (PMA), to more closely resemble Kupffer cells, the liver-resident macrophages (Granitzny et al., 2017).

Indirect co-culture often utilizes Transwell systems. In this case, HepG2 cells are cultured in the lower compartment while THP-1 cells (often differentiated with PMA) are seeded in an upper insert. The upper insert is separated from the lower compartment by a porous membrane. This allows the exchange of soluble factors without direct cell–cell contact (Padberg et al., 2021). To study the impact of gut–liver crosstalk on nutrient absorption, drug metabolism and the development of MASLD, Transwell systems can be designed using HepG2 cells and Caco-2 intestinal epithelial cells (Meroni et al., 2022).

Alternatively, hepatocytes can be co-cultured with immune cell supernatant to study immune-liver interactions, particularly in contexts such as inflammation, infection, drug-induced liver injury or fibrosis. This setup is more controlled than combining two or more different cell types using soluble factors only without direct cell–cell contact. Consequently, the system is less variable (there is no overgrowth of immune cells or unexpected interactions) and the observed effects are easier to attribute to secreted signals. However, this model lacks direct dynamic cell–cell interactions between immune cells and hepatocytes. The main limitations of 2D systems are their inability to replicate of three-dimensional (3D) architecture and their rapid loss of cell function. To address more complex issues or better replicate the *in vivo* situation, 3D models are often preferable.

## 3 Three-dimensional (3D) models

### 3.1 Spheroids

Spheroids are 3D cell aggregates formed through self-organized cell–cell adhesion. They differ fundamentally from traditional 2D cell cultures and can be generated from various cell types, such as tumor cells, epithelial cells and stem cells. They form when cells sequester their own secreted ECM components and proteins to self-assemble into compact, spherical configurations. This process is induced by culturing cells under non-adherent conditions, such as hanging drop cultures, low-attachment plates, spinner flasks, or microfluidic devices. These conditions prevent cells from spreading out on a flat substrate and instead promote cell–cell interactions (Cacciamali et al., 2022; Baumert et al., 2025).

In a typical spheroid, the architecture creates microenvironments that influence differentiation, proliferation, and metabolic activity, thereby enhancing the physiological relevance of *in vitro* studies. But it should be kept in mind that cells at the periphery are usually more proliferative due to their better access to nutrients and oxygen. Cells in the center encounter oxygen, nutrient, and metabolite gradients that can lead to a hypoxic core not similar to *in vivo* tissues (Kunz-Schughart et al., 2004; Velliou et al., 2024). These 3D systems construct cell–cell and cell–ECM interaction networks, which play a significant role in various cellular mechanisms and subsequently maintain the cellular properties (Rosso et al., 2004; Cushing and Anseth, 2007). They promote the expression of stemness markers, and often exhibit increased resistance to external influencing factors, such as drugs or toxins (Zhang et al., 2012). This makes them particularly valuable for studying tumor biology, drug testing, for toxicological studies and regenerative medicine (Kunz-Schughart et al., 2004).

Despite these advantages, there are also several limitations of spheroids. The technical challenges involved in constructing and maintaining uniform spheroids, particularly with respect to achieving reproducibility and uniformity across samples, are complex. To create a reproducible and sophisticated model, it is essential to validate the following parameters: the combination of cells, the size of the spheroid, the composition of the culture medium, and the incubation time (Tevlek et al., 2023). Variability in spheroid size and structure can lead to heterogeneous experimental outcomes. The formation of oxygen and nutrient gradients within the aggregates can lead to hypoxia and cell death in the core of the spheroid, which complicates standardization and influences experimental outcomes (Cacciamali et al., 2022). In addition, analytical methods are more demanding as dyes and antibodies often struggle to penetrate the interior of the spheroid, making detailed analysis more challenging.

Moreover, while spheroids replicate many features of native tissues, they generally lack complete vascularization and immune system components. These are critical for establishing long-term tissue viability and for studying complex interactions that occur *in vivo*. Researchers have attempted to overcome these limitations by integrating spheroids with microfluidic systems or co-culture models that include endothelial cells or immune cells to better simulate *in vivo* conditions (Velliou et al., 2024; Kim et al., 2024). The lack of a real extracellular matrix also restricts the physiological relevance of some processes (Cacciamali et al., 2022; Rosso et al., 2004).

#### 3.1.1 Gastroenterology

Spheroids represent a particularly efficient and cost-effective approach in the field of gastroenterology. The model is more complex than its 2D analogues, though at the same time it is simpler than organoids. Epithelial spheroids can be developed from patients’ biopsies, pluripotent stem cells or from cell lines, such as Caco-2 or HT-29 cells (VanDussen et al., 2015; Scharl et al., 2013). Spheroids are frequently utilized in preclinical cancer research, infectious diseases research, disease modelling, tissue engineering and tissue regeneration. Furthermore, spheroids fabricated from patients’ biopsies represent a valuable tool for cell biobanking, personalized assays and medicine (Rescigno, 2015). As cancer spheroids exhibit histological features and gene expression similar to those of the original tumor tissue, they may be useful in cancer research and personalized drug screening (Zhang et al., 2023).

Spheroids can be created from 1 cell type, forming homotypic spheroids, or from two or more cell types, to form multicellular spheroids (Tevlek et al., 2023; Sant and Johnston, 2017). The development of the micropatterned gut spheroid generator by Fu and Shao paved the way for the production of spheroids from human pluripotent stem cells via mechanically enhanced tissue morphogenesis (Lin et al., 2023). The generator promotes the formation of various region-specific types of gut spheroids.

Epithelial cells exhibit the capacity for self-assembly into the spheroids, whether as free-floating forms or during culturing on ECM. It is important to note that ECM enhances the differentiation of epithelial cells in comparison to growing cells on plastic. The role of the ECM in the differentiation of epithelial cells and cell growth is determined by the presence of laminins, collagens, glycoproteins and proteoglycans (Ashkenas et al., 1996; Hughes et al., 2010a). The most widely used commercially available ECM is Matrigel, which is isolated from mouse sarcoma (Hughes et al., 2010b). At the same time, synthetic hydrogels composed of poly(2-alkyl-2-oxazoline) can be considered as a potential alternative to Matrigel (Vanhoeijen et al., 2025). In a recent study, Bianchi et al. demonstrated the potential of gelatine methacrylate microgel as a spherical scaffold for the creation of reverse-polarity spheroids from HT-29 cells (Bianchi et al., 2025). This approach has the capacity to recreate the epithelial barrier. Additionally, the size of the spheroids is controlled with a high degree of reproducibility. Furthermore, spherical gelatine scaffold prevents core necrosis, which frequently occurs in conventional intestinal spheroids.

In a recent study, Samy et al. successfully demonstrated the effectiveness of their method, sacrificial micromodling technique, in precisely controlling the geometry of Caco-2 spheroids. This method facilitates cell differentiation within only 6 days (Samy et al., 2019; Cerchiari et al., 2015).

Recently, Kraski and colleagues used ultra-low attachment plates to develop multicellular intestinal spheroids from primary fibroblasts, Caco-2, goblet cells (HT-29-MTX-E12) and activated monocytes (Kraski et al., 2024). The diameter of these multicellular spheroids is approximately 505 µm. The characterization of the spheroids was conducted by employing scanning electron microscopy, transmission electron microscopy and immunofluorescent staining. It is noteworthy that, in contrast to the 2D Caco-2 model, where cells require 21 days to differentiate, the Caco-2 cells in the present model developed microvilli after 2 days. While the expression of sodium-dependent glucose cotransporter 1 (SGLT1) was found to be significantly increased, the expression of peptide transporter 1 (PEPT1) was significantly reduced in comparison with the 2D Caco-2 model. The authors hypothesized that an increased level of epidermal growth factor from fibroblasts may potentially affect the downregulation of PEPT1. The multicellular model exhibited a robust approach as a functional assay to investigate intestinal campylobacteriosis. Multicellular spheroids can be considered a more reliable model in comparison to spheroids formed by a single cell type.

Michiba et al. demonstrated that spheroids from human jejunal tissue are a useful tool for the recreation of epithelial cells and the prediction of drug absorption. Furthermore, cells derived from these spheroids have been found to express enzymes such as CYP3A, CYP2C9, carboxylesterase 2, as well as uptake and efflux transporters including PEPT1, P-glycoprotein, and breast cancer resistance protein. These enzymes and transporters are crucial for predicting the rate of drug absorption (Michiba et al., 2022). Recently, the same group validated their approach with terminal ileum. Overall, the ability to use authentic, region-specific intestinal epithelium is crucial for studying drug absorption and metabolism (Michiba et al., 2025).

It is important to remember that the model has limitations. The main and most common limitation of all spheroid models is the challenge of controlling their size. Another limitation is necrosis of cells due to a lack of oxygen in the center of the spheroids (Fitzgerald et al., 2015). Furthermore, to enhance the application of spheroids, the model requires validation though standardization, harmonized protocols, and reproducibility assessment. In addition, it is important to note that spheroids only represent the *in vivo* situation of the real intestine to a limited extent. In general, multicellular spheroids are a more robust model than spheroids formed from one cell type. Epithelial cells in spheroids demonstrate faster and more efficient differentiation than in 2D models. Therefore, intestinal spheroids could be useful tools for drug screening and disease modeling.

#### 3.1.2 Hepatology

Traditional *in vitro* models such as 2D cell cultures fail to replicate the human liver’s complex multicellular interactions, ECM dynamics, and metabolic gradients. 3D liver spheroids aggregate primary hepatocytes and NPCs into organotypic spheres that better mimic *in vivo* conditions. These structures promote enhanced cell-cell and cell-ECM contacts, essential for sustaining liver-specific functions like albumin production, CYP450 metabolism, and bile acid synthesis (Cui et al., 2025; Haag et al., 2025; Bonanini et al., 2022).

In hepatological research, liver spheroids are used to study liver toxicity, drug metabolism and disease models such as MASLD. Their use allows to monitor and manipulate complex cellular behaviors including lipid accumulation, inflammatory responses, and fibrotic signaling within a controlled and reproducible framework (Baumert et al., 2025; Youhanna et al., 2025).

Patient‐specific liver spheroids from induced pluripotent stem cells (iPSCs) or primary cells eliminate discrepancies by providing a human-relevant, scalable *in vitro* system that supports both acute and chronic studies (Youhanna et al., 2025). The integration into multiplexed assays, enabling the parallel evaluation of a huge number of compounds for anti-steatotic, anti-inflammatory, and anti-fibrotic effects enhances the predictive accuracy of such systems for drug-induced liver injury and metabolic responses. With regulatory agencies increasingly supporting the replacement of animal testing with human-relevant *in vitro* models, spheroids are at the forefront of next-generation platforms for preclinical studies and personalized risk assessments in e.g., MASLD (Cui et al., 2025; Youhanna et al., 2025).

An increasing amount of research has included liver spheroids in multi-level translational frameworks to clarify the mechanistic basis of MASLD. For example, Baumert et al. used human liver spheroids within an integrated spheroid-to-population framework to study the molecular basis of MASLD caused by environmental contaminants, such as perfluoroheptanoic acid (PFHpA) (Baumert et al., 2025). In this study, liver spheroids were exposed to non-cytotoxic concentrations of PFHpA for several days. This led to measurable increases in lipid accumulation and transcriptomic alterations that reflect the pathophysiology of MASLD. These findings have facilitated the identification of critical dysregulated metabolic pathways, such as innate immunity, lipid metabolism, and inflammatory signaling, that are also observed in clinical settings (Baumert et al., 2025). Further, the use of imaging techniques in spheroid assays, including Nile Red and 4′,6-diamidino-2-phenylindole (DAPI) staining combined with confocal microscopy, has allowed for detailed quantification of intracellular lipid deposits, a hallmark feature of MASLD. Single-cell RNA sequencing on dissociated spheroids enables the dissection of cell type-specific transcriptional responses following toxicant exposure, thereby revealing intercellular communication patterns critical for disease progression (Baumert et al., 2025). These findings underscore the spheroid model’s capability in bridging the mechanistic gap between *in vitro* experiments and *in vivo* human pathology, enhancing our understanding of MASLD’s progression from simple steatosis to advanced steatohepatitis (MASH).

The successful generation and maintenance of liver spheroids require optimized protocols that ensure physiologically relevant cell ratios, reproducible architecture, and sustained functionality over extended periods. Kim et al. describe a method to isolate primary human hepatocytes along with NPC types such as Kupffer cells and hepatic stellate cells (HSC) and co-culture them in ultra-low attachment microplates, leading to spontaneous spheroid formation within days (Kim et al., 2024). The use of specific mixtures containing free fatty acids, sugars, lipopolysaccharide (LPS), and transforming growth factor-β 1 (TGF-β1) are used to induce metabolic stress that mimics conditions seen in MASLD. These spheroids are then assessed biochemically and via imaging for markers of liver injury, lipid accumulation, and fibrosis, allowing for studies on both induction and regression of liver pathology when the disease-inducing stimuli are removed (Kim et al., 2024).

The future direction of spheroid research in hepatology involves integrating multi-omics data. Combining transcriptomic, proteomic and metabolomics profiling of spheroids exposed to conditions that induce MASLD enables researchers to develop a systems-level understanding of the disease mechanisms underlying lipid dysregulation and fibrogenesis. This integrative approach improves mechanistic insight into the model and aids the identification of novel biomarkers and therapeutic targets that could lead to clinical interventions (Baumert et al., 2025; Zhao et al., 2025).

The continuous refinement of cellular composition within spheroid cultures, including the incorporation of additional liver cell types such as liver sinusoidal endothelial cells (LSECs) and immune cells, is expected to enhance their physiological relevance. Such multicellular spheroid systems would better capture the heterogeneity and intercellular communication that are essential to the pathogenesis of MASLD, thereby improving the model’s utility in drug screening and toxicity testing (Kim et al., 2024; Bonanini et al., 2022).

Despite their significant advantages, there are inherent limitations to liver spheroids that must be addressed to maximize their potential in MASLD research. One of the primary challenges is the issue of nutrient and oxygen diffusion, which can result in the formation of a necrotic core in larger spheroids. While the small size of spheroids in many high-throughput applications often circumvents this issue, further optimization is required for long-term culture studies and modeling advanced fibrosis, where spheroid dimensions may increase (Kim et al., 2025a). Additionally, while spheroids faithfully mimic several aspects of the liver microenvironment, they generally lack certain tissue-specific features such as a functional vasculature and an intact immune component. These factors can limit their ability to fully replicate the *in vivo* conditions associated with MASLD, particularly in studies that require detailed analysis of inflammatory cell recruitment and hepatocyte NPCs crosstalk (Baumert et al., 2025; Kim et al., 2025b). Consequently, there are ongoing efforts to combine spheroid models with microfluidic platforms to create hybrid systems, such as liver-on-chip devices, which improve nutrient perfusion and better emulate the dynamic *in vivo* environment. These integrated platforms are expected to enhance the predictive accuracy of *in vitro* models and facilitate the study of chronic liver injury responses (Velliou et al., 2024; Morisseau, 2026).

### 3.2 Organoids

Organoids replicate many of the structural and functional characteristics of whole organs by taking advantage of the self‐organization properties of stem cells (Almeqdadi et al., 2019). These systems are generated by isolating tissue-specific or pluripotent stem cells and embedding them in a supportive ECM which contrasts with spheroids which usually are derived from cancer cells. This matrix provides a biochemical and biophysical surrounding of the native tissue niche, enabling the cells to proliferate, differentiate and arrange themselves into miniaturized tissue structures (Lehmann et al., 2019). Compared to 2D models, organoids offer a far more physiologically relevant model by preserving important tissue architectures, gene expression patterns, and functionality (Andrews and Kriegstein, 2022). Their development has transformed multiple areas of biomedical research by providing a link between basic cellular assays and whole organism models, enabling research about development, disease and therapy in a human-specific context (Bar-Ephraim et al., 2020).

Over the past decade, advances in biomaterials design, particularly the use of hydrogels such as Matrigel that mimic essential ECM components, have greatly improved reproducibility and functionality. In addition, the performance of organoids has been further enhanced by refined culture media formulations enriched with specific growth factors and signaling molecules (Li et al., 2022). Furthermore, genetic engineering technologies such as CRISPR/Cas9 allow precise manipulation of cellular constituents in all *in vitro* model systems. However, in organoids, the study of gene function and the modelling of specific disease mutations can be conducted in a three-dimensional (3D) environment that is more physiologically relevant (Choi et al., 2023).

To enhance the application of organoids, in 2023 the Ministry of Food and Drug Safety of South Korea established the Organoid Standards Initiative and developed general guidelines for organoid manufacturing and quality evaluation. Ahn et al. presented a detailed report on the essential performance criteria for organoid development (Ahn et al., 2024; Ahn, 2024).

#### 3.2.1 Gastroenterology

3D intestinal organoids, also known as ‘mini-intestines’, are a self-organized, three-dimensional composition of cells specific to the intestine. To a certain extent, organoids represent the uniqueness and heterogeneity of intestinal cells *in vitro*, and can replicate specific organ functions (Lancaster and Knoblich, 2014; Taelman et al., 2022; Bao et al., 2023). While IBD is a complex chronic inflammatory disease, stem cell-derived intestinal organoids can be used to investigate epithelial barrier function and immune responses in IBD. Moreover, patients-derived intestinal organoids represent a powerful tool to explore the still unclear etiology of IBD and to evaluate potential therapeutic options (Tian et al., 2023). Intestinal organoids represent the earliest type of human organoids. Since 2011, these types of organoids have undergone continuous improvement. Intestinal organoids reflect the heterogeneity and architecture of human tissues. Furthermore, they can function as a valuable resource for research in the field of human intestinal physiology. The formation of these 3D structures involves the utilization of primary tissues, adult stem cells, iPSCs, or embryonic stem cells (ESCs) via a process of cell sorting and spatially restricted lineage differentiation (Lancaster and Knoblich, 2014; Rauth et al., 2021; Corsini and Knoblich, 2022). Stem cells isolated from the basement membrane of intestinal crypts can self-renew and differentiate into enterocytes or colonocytes due to their ability to undergo differentiation. It is important to note that intestinal epithelial cells require the support of the basement membrane for cell differentiation (Hahn et al., 1990). Thus, the seeding of cells on a matrix is essential, with options including Matrigel, collagen or synthetic hydrogels. The cells are then cultured in a medium containing Wnt3a, fibroblast growth factor 4, R-spondin, noggin and epithelial growth factor which are important components of the intestinal stem cell niche (Lancaster and Knoblich, 2014; Dutton et al., 2019; Zhao et al., 2022).

3D organoids are a more precise model of human tissue physiology compared to animal or 2D cellular models. Intestinal organoids are particularly valuable for investigating intestinal function and disease phenotypes, modelling human intestinal development, and screening drugs (Lancaster and Knoblich, 2014; Taelman et al., 2022; Tian et al., 2023; Spence et al., 2011).

One of the advantages of using gut organoids is that they can be used to model diseases or healthy physiological processes as an alternative to animal models. This includes modelling infectious diseases, intestinal fibrosis, cancer, drug testing, nutrient transport and nanomedicine (Lancaster and Knoblich, 2014; Bao et al., 2023; Banerjee et al., 2024; Barker et al., 2010; Fordham et al., 2013; Watson et al., 2014; Finkbeiner et al., 2015). In addition, it could be a useful tool for personalized treatment assays (Angus et al., 2019). However, there are some limitations, as a gut organoid generally cannot sufficiently recapitulate immune cells and a functional gut microbiome. There is also a lack of mesenchymal heterogeneity, lamina propria, vascular and neuronal structures (Taelman et al., 2022; Bao et al., 2023). While intestinal organoids are characterized by the lumen inside of the structure, the access to the apical side is reduced, facing the limitation of potential drug screening, studies with microbiome, etc. Nevertheless, some recent studies are focusing on establishing of organoids with a reversed apical side (an apical-out organoid model) (Taelman et al., 2022; Co et al., 2019; Co et al., 2021; Li et al., 2020). Currently, organoid technology remains associated with high costs and labor-intensive procedures. The implementation of automatization and further standardization will be essential to improve the scalability and application of this methodology (Ge et al., 2024).

#### 3.2.2 Hepatology

In hepatology, organoids have begun to emerge as an indispensable model that overcomes the limitations of animal studies, which are often affected by interspecies differences, and conventional 2D hepatocyte cultures, which lose key liver-specific features such as polarity and metabolic function (Liu et al., 2022). Liver organoids are obtained from hepatic progenitors, primary hepatocytes or pluripotent stem cell derivatives in conditions that induce liver-specific differentiation and structural organization. These systems are crucial for accurately modeling liver biology, as they maintain essential liver functions, including the production of albumin, drug metabolism, glycogen storage and bile canaliculi formation (Kim et al., 2025b). Their capacity for self-renewal and expansion over extended periods while maintaining genomic stability, establishes them as highly promising platforms for basic research and the development of novel therapeutic strategies (Han et al., 2023).

The clinical significance of liver organoids is particularly evident in the context of MASLD (Osonoi and Takebe, 2024). They provide a highly controllable and manipulable tool for modeling patient-specific genetic determinants, cellular heterogeneity, and tissue microenvironments with a high level of accuracy compared to the human liver *in vivo* (Obeid D. A. et al., 2024). A major advantage of using organoids in MASLD research is the ability to create personalized liver models that accurately reflects the genomic and phenotypic characteristics of the disease. For instance, induced iPSC-derived liver organoids can be developed from individuals harboring specific MASLD-associated single nucleotide polymorphisms, such as those in the patatin-like phospholipase domain-containing protein 3 (PNPLA3) or glucokinase regulator (GCKR) genes and then be used to study the underlying mechanistic of lipid accumulation, impaired metabolic signaling, and inflammatory responses (Osonoi and Takebe, 2024). These systems allow for the large-scale, CRISPR-based screening of loss of function to identify steatosis-protective genes and genetic variants that influence risk of MASLD. This advances our understanding of the polygenic nature of the disease and its interindividual variability (Osonoi and Takebe, 2024).

Furthermore, liver organoids providing a more accurate model of human drug responses than conventional hepatocyte cultures (Ju et al., 2024). Therapeutic interventions of MASLD must navigate complex metabolic networks and minimize off-target effects. Organoids enable the high-throughput screening of candidate compounds in a controlled environment that incorporates the interactions of multiple levels between hepatocytes, cholangiocytes and NPCs (Harrison et al., 2021). In addition, incorporating NPCs within liver organoid co-culture systems provides a more comprehensive representation of the liver microenvironment (Jalan‐Sakrikar et al., 2023). These multi-lineage organoids replicate hepatocyte functionality and model the crosstalk between different cell types that drives pathological evolution from benign steatosis to a fibrotic steatohepatic state. Furthermore, such models are invaluable for exploring the dynamics of immune cell infiltration and inflammatory signaling, which are crucial for the onset and progression of MASH (Osonoi and Takebe, 2024).

Organoid-guided precision hepatology leverages the concept of ‘donor-mosaic’ organoid models, incorporating cells from multiple patients into a single organoid culture system to capture the genetic variability observed in human populations. This strategy enables the analysis of disease susceptibility across different genetic backgrounds and helps identify subgroup-specific therapeutic targets and biomarkers (Osonoi and Takebe, 2024). Another important aspect that highlights the significance of liver organoids in hepatology is their use in regenerative medicine. Traditional liver transplantation is hindered by shortages of organs and the risk of immune rejection, while the use of primary human hepatocytes is limited by difficulties in maintaining their viability and ability to grow in a laboratory setting (Table 2) (Liu et al., 2022). Due to their characteristic capacity for self-renewal and expansion, coupled with their ability to recapitulate liver-specific functions, liver organoids present a promising alternative for cell-based therapies (Jalan‐Sakrikar et al., 2023).

Preclinical studies have demonstrated that transplanted liver organoids can engraft into diseased liver tissue, restore liver function and integrate with the host vasculature. This offers a potential therapeutic strategy for chronic liver diseases (Han et al., 2023; Takebe et al., 2012). These regenerative approaches are further supported by gene-editing capabilities, which allow genetic mutations that cause liver dysfunction to be corrected prior to therapeutic transplantation. This combines the benefits of personalized medicine with regenerative therapy.

Despite these notable advancements, challenges remain in liver organoid research, particularly in fully recapitulating the liver’s complex architecture *in vitro*. Key limitations include the incomplete representation of hepatocyte zonation, which is a critical feature that governs metabolic heterogeneity and the difficulty of incorporating functional vascular, immune and neural components into organoid models (Osonoi and Takebe, 2024). Furthermore, incorporating NPC types into liver organoids is essential for modelling the multicellular interactions that underpin MASLD progression. Kupffer cells, HSC and liver sinusoidal endothelial cells all play a crucial role in mediating inflammation, fibrogenesis and nutrient exchange in the liver (Jalan‐Sakrikar et al., 2023). Recent studies have begun to integrate these cell types into organoid cultures, providing a more complete platform for studying the interaction between parenchymal and non-parenchymal compartments (Harrison et al., 2021).

### 3.3 Scaffold-based 3D model

The principle of 3D scaffold models is based on the utilization of physical supports for growth and migration, mimicking an extracellular environment that is crucial for the investigation of disease mechanisms, disease prognosis and drug resistance. Compared with spheroids and organoids, 3D scaffold models represent a more advanced model and provide a more physiologically relevant platform. However, there are still important limitations and challenges that currently limit its commercial application.

The concept of a scaffold-based 3D model is widely used, particularly in cancer research for culturing liver cancer cells and investigating colorectal cancers, as well as for tissue regeneration and drug screening. The advantage of a scaffold-based 3D model over a 2D model is that it allows cells to recreate the architecture and microenvironment of the tissue. The complexity of tumor tissue comprising cancer cells, immune cells, fibroblasts, blood vessels, and ECM, is crucial for understanding metastasis progression, epithelial-to-mesenchymal transition, drug resistance and prognosis. Thus, a model, that mimics these parameters is essential for properly investigating cancer. There are several types of scaffold-based models. Polymer-based scaffolds made of natural compounds as collagen, gelatin, chitosan, or synthetic polymers as polyethylene glycol, polyglycolic acid, and polycaprolactone. Additional scaffold sources include hydrogels, as well as decellularized tissue scaffolds and hybrid scaffolds (Zhu et al., 2023; Kadam et al., 2025; Abuwatfa et al., 2024).

Recreation of complex tissue architecture is important not only for disease investigation, but also for studying host-microbiome interactions. Thus, a 3D polymeric scaffold model using Caco-2 cells was e.g., implemented to investigate the interactions of probiotics with cancer cells (Costello et al., 2014). Roh and colleagues used 3D silk scaffolds seeded with human colonoids and monocyte-derived macrophages as a model to investigate the therapeutic options for patients with IBD (Roh et al., 2019). An example of hydrogel-based scaffold model was presented by Tselekouni et al. An alginate-gelatine hydrogel was used to investigate epithelial-stromal interactions in the gut. They utilised HT-29 intestinal epithelial cells and IMR-90 fibroblasts (Tselekouni et al., 2026).

An important application of the 3D scaffold model was demonstrated by D’Angelo and colleagues. The group of Urbani and Agostini developed a model to study colorectal cancer liver metastasis using patient-derived scaffolds. They established a decellularization protocol for patient-derived colorectal cancer liver metastasis to produce an extracellular matrix without cells. These 3D scaffolds were subsequently colonized with HT-29 cells. This 3D scaffold model can be implemented for drug screening, cytotoxicity assays, and prognostic studies. Moreover, the drug resistance observed in this model closely reflects the characteristics of *in vivo* cancer. However, the main limitation of this model is the variability among patients and their cancer extracellular matrices (D’Angelo et al., 2020). Another example of the decellularization technique was presented by the group of Chen, who utilized a 3D decellularization human liver scaffold to investigate the Hepatitis B virus. By seeding these scaffolds with primary human hepatocytes and HepG2-NTCP cells, they created a model that supported a prolonged infection period. This model provides a valuable platform for studying viral pathogenesis and screening antiviral drugs (Zha et al., 2019).

A 3D scaffold model derived from human cirrhotic liver is an important tool to investigate the alteration of liver in progression of hepatocellular carcinoma. It has been shown that cirrhotic liver extracellular matrix promotes TGF-β1 epithelial mesenchymal transition (Mazza et al., 2019; Caon et al., 2024).

It is important to mention that there are scaffold-free 3D cell models when cells assembled into 3D structures without external scaffold. For example, Banavar et al. described the simplified scaffold-free 3D aggregation techniques, such as the scraping-induced cell clump method. Using this low-cost 3D method, the formation of viable 3D periodontal-ligament-derived mesenchymal stem cells-ECM clumps without specialized scaffolds or microfabrication was demonstrated. While the density of cells can be higher in such methods compare to 3D scaffolds models, there are some limitations as uncontrolled size, shape and limited reproducibility (Banavar et al., 2021).

The biggest limitation of 3D scaffolds models is reproductivity. Due to the presence of scaffold, it is challenging to control pH, temperature and access to the growth factor molecules. Furthermore, due to limited standardization, this model is currently not applicable for commercial or clinical use. Essential steps are required for the implementation of GMP principles and the harmonization of protocols (Abuwatfa et al., 2024).

## 4 Organ-on-chip models

Organ-on-chip models are microfluidic culture models of human organs, including e.g., the liver and intestine. Typically, these models comprise multiple cell types. However, the most significant distinction between organ-on-chip and alternative models is the presence of fluid flow and peristalsis-like motions (Özkan et al., 2024). Organ-on-chip models can be classified into two main categories: single organ-on-chip models and multi-organ-on-chip models. For example, recent work by Lucchetti and colleagues has implemented a multiorgan-on-chip model (e.g., gut-and liver-on-chip) for the purpose of investigating drug metabolism along the gut–liver axis (Lucchetti et al., 2024; Bhatia and Ingber, 2014).

The European Commission’s Joint Research Centre created the CEN-CENELEC Focus Group that published an Organ-on-Chip Standardization Roadmap in 2024. In the same year, the International Organization for Standardization (ISO) established a Subcommittee on Microphysiological systems and Organ-on- Chip „ISO/TC 276/SC 2”. Further, the FDA announce the intention to replace animal models in the preclinical stage of novel drug investigation with organ-on-chip technology, organoids, and artificial intelligence by 2030. However, currently there are several challenges regarding full integration of these novel methods in pharmaceutical laboratories. There is variability between chips due to inconsistent origin of cells used on the chip. Different donors for human primary cells, as well as the use of cell lines or induced pluripotent stem cells and different protocols for cell differentiation may lead to variability in results between institutes. To be accepted by regulatory authorities, the model must be robust, reproducible, standardized and validated.

While alternative models can provide valuable data for drug development, screening, and potential toxicity assessment, conventional animal models provide information regarding systemic toxicity in the whole body. In addition, certain toxic effects may not be detected when a component becomes toxic only after hepatic metabolism. The biggest limitation for organoids as a tool for personalized drug screening for patients with cancer is the time required to grow a sufficient number of cells and the static conditions of the system (Ingber, 2026; Bisconti et al., 2025).

### 4.1 Gut

In contrast to general intestinal organoids, a gut-on-chip model is a continuously perfused three-dimensional model comprising epithelial, endothelial, and immune cells, which creates a more complex structure. As a result, a gut-on-chip model better mimics the physiology of the intestine in comparison with 2D and 3D cell models (Bhatia and Ingber, 2014; Maurer et al., 2019; Thomas et al., 2023). Typically, the chip is a two-compartment model; however, three co-laminar channels are also present as a HuMiX model (human–microbial crosstalk) (Thomas et al., 2023; Shah et al., 2016). To develop an intestinal barrier on a chip, a variety of cell types may be employed, including Caco-2 cells, human pluripotent stem cells, adult stem cells, human colon tissue, or intestinal organoids (Siwczak et al., 2021). The intestinal barrier can be cultured on a flexible semipermeable membrane coated with an extracellular matrix or hydrogel column that closely resembles the *in vivo* environment (Nguyen et al., 2024; Kim et al., 2012; Trietsch et al., 2017). The opposing chamber of the chip is seeded with human monocytes (THP-1 cells or primary cells), along with human umbilical vein endothelial cells (HUVEC) or human intestinal microvascular endothelial cells (Lucchetti et al., 2024; Thomas et al., 2023; Kasendra et al., 2020). The culturing of cellular layers may be performed on a multi-organ-tissue-flow (MOTiF) biochip, a MultiU-Int microfluidic chip, or an alternative device (Nguyen et al., 2024; Raasch et al., 2015). The most common materials for the fabrication of membranes are polydimethylsiloxane, polycarbonate, polyester, polyethylene terephthalate, and polytetrafluoroethylene (Thomas et al., 2023). The chip device can be fabricated from polycarbonate or polydimethylsiloxane. In a study conducted by Henry and colleagues, a microchip model with integrated electrodes was developed for the non-invasive monitoring of TEER in real time (Henry et al., 2017).

To establish a continuous flow of the culture medium (0.5–50 μL/min, shear stress: 0.01–0.07 Pa), a peristaltic pump is employed (Maurer et al., 2019; Kasendra et al., 2020; Shin et al., 2019; Sontheimer-Phelps et al., 2020; Wang et al., 2023). The perfusion helps to extend the lifetime of the cells (Alonso-Roman et al., 2024). Furthermore, it was demonstrated that fluid shear stress on the apical part of Caco-2 cells is essential for cellular differentiation (Delon et al., 2019). Shear stress, oxygen gradient, and cyclic strain represent critical parameters in the establishment of a gut-on-chip model (Thomas et al., 2023). Moreover, these conditions enable to mimic the physiological shear stress on the cell surface and mimic the fluid flows within the human capillaries and intestinal lumen (Bein et al., 2018). In general, Caco-2 cells (1.2–3.0 × 10^6^ cells/mL) are seeded on one side of the membrane, while HUVEC cells are seeded on the opposite chamber at a density of 1.2–2.6 × 10^6^ cells/mL (Lucchetti et al., 2024; Feile et al., 2024).

Recently, Mitrofanova et al. combined organoids and organ-on-chip technologies to create a bioengineered mini-colon model. This model displays morphological similarities to native tissue and exhibits crypt topography and functional characteristics that facilitate the reproduction of homeostatic cell turnover and differentiation (Mitrofanova et al., 2024). By employing a microchannel that resembles the microstructure of a human colonic crypt, the authors were able to achieve cell diversity. Specifically, they were able to cultivate transit-amplifying cells, stem cells, colonocytes (early, mature, crypt-top, and absorptive), goblet and enteroendocrine cells (Mitrofanova et al., 2024; Nikolaev et al., 2020). In a recent study, Kasendra and colleagues successfully developed a human duodenum intestine-chip model using organoids and the organ-on-chips technology. This model is suitable for the assessment of drug transport, metabolism, and drug-drug interactions (Kasendra et al., 2020; Kasendra et al., 2018).

The gut-on-chip model has a number of potential applications in personalized medicine, pharmacological and toxicological analysis, and the investigation of host-microbial interactions and immunopathogenesis in IBD (Maurer et al., 2019; Nguyen et al., 2024; Alonso-Roman et al., 2024; Morelli et al., 2023; de Haan et al., 2021; Malaguarn et al., 2021). An important application of the gut-on-chip model is the investigation of interactions between fecal microbiota transplantation and patient biopsy-derived cell, enabling the personalised evaluation of therapeutic strategies for IBD (Kaden et al., 2025). The advantages of organ-on-chip models include their flexibility in terms of cell composition, the ability to conduct long-term experiments due to the dynamic nature of the system, and the capacity for independent investigation of different compartments (Alonso-Roman et al., 2024). It is important to note that while the gut-on-chip model is a complex system that can simulate the physiology of the tissue in some ways, it is not a comprehensive model (Kang et al., 2021). The limitation of this model is its relatively small volume and limited size. Furthermore, it is currently not feasible to regulate the microenvironment and cellular conditions at the scale-up level. In addition, to obtain reliable results, it is essential to standardize and validate organ-on-chip models (Alonso-Roman et al., 2024; Obeid P. J. et al., 2024).

### 4.2 Liver

Liver-on-chip systems integrate microfluidics, biomaterials and cell biology to engineer an *in vitro* microenvironment that mimics the complex *in vivo* hepatic niche (De Chiara et al., 2021). These models use 3D cell cultures incorporating primary hepatocytes, differentiated cell lines, hepatic stellate cells, Kupffer cells and endothelial cells to mimic the liver sinusoid. In this model, controlled gradients of oxygen, nutrients and metabolites are generated to effectively reproduce liver zonation. This is a critical aspect of hepatic metabolism and functional heterogeneity (Polidoro et al., 2021; Hassan et al., 2020). In essence, liver-on-chip platforms address the need for systems that can maintain liver-specific functions over extended periods while allowing real-time monitoring and dynamic control of the microenvironment (Messelmani et al., 2022). Underlining how important the microenvironment is for cells, Parker et al. showed that liver cells multiplied and their metabolic activity was increased when placed together with mesenchymal cells from human placenta (Parker et al., 2008). Advanced fabrication techniques, including soft lithography and 3D bioprinting, have enabled the development of sophisticated liver-on-chip models that can recreate liver lobule architecture and complex cellular interactions (Polidoro et al., 2021). This multicellular arrangement is important for studying drug metabolism and hepatotoxicity because it allows cell-cell interactions that drive disease progression and drug responses to be investigated (Velliou et al., 2024).

Using liver-on-chip models for drug screening provides a more predictive method of assessing hepatotoxicity and therapeutic efficacy than traditional static cultures or animal models (Baudy et al., 2020). These systems can evaluate the safety profiles and pharmacokinetics of candidate drugs by accurately modeling hepatic metabolism and drug-induced liver injury (Hassan et al., 2020). Conducting high-throughput screening on these chips accelerates the early stages of drug development by identifying promising candidate compounds and reducing dependency on animal testing (Polidoro et al., 2021). Furthermore, integrating pharmacodynamic and pharmacokinetic analyses into these models enables the prediction of potential drug–drug interactions and toxicity issues under pathophysiological conditions (Rezvani et al., 2023). These predictive capabilities are crucial in reducing failures in late-stage clinical trials, ultimately streamlining the drug development pipeline and ensuring greater translational relevance (Hassan et al., 2020).

Liver-on-chip models have successfully simulated MASLD progression (Morisseau, 2026; Hassan et al., 2020). Furthermore, the dynamic conditions created by microfluidic perfusion facilitate the gradual accumulation of lipids in a manner that closely resembles chronic disease progression. This makes liver-on-chip models a more accurate representation of MASLD than static cultures (Cui et al., 2025). A significant advantage of liver-on-chip platforms in the context of MASLD is their ability to integrate patient-specific cells, including iPSC-derived hepatocyte-like cells, which capture the genetic and metabolic heterogeneity observed in human populations (Xia et al., 2024). Such patient-specific models have the potential to advance precision medicine by predicting how individuals will respond to anti-steatotic and anti-inflammatory therapeutics (Rezvani et al., 2023). In addition, gene editing tools and advanced omics techniques have been used alongside liver-on-chip systems to shed light on the molecular mechanisms underlying MASLD. This includes investigating the impact of key genetic variants, such as PNPLA3 I148M, on lipid metabolism and inflammatory responses (Cui et al., 2025).

A critical feature of liver-on-chip models is the ability to perform real-time monitoring of various biochemical parameters using integrated biosensing technologies (Messelmani et al., 2022). These biosensors enable the dynamic measurement of oxygen consumption, glucose uptake, lactate secretion and levels of specific biomarkers, such as albumin and CYP450 enzymes, all indicators of hepatic function (Hassan et al., 2020). By capturing these functional readouts, researchers can correlate the physiological status of liver tissue with pathological changes induced by drugs or metabolic stress (Polidoro et al., 2021). Recent advances in organ-on-chip technology have enabled multiple organ systems to be integrated onto a single chip, thereby facilitating the study of inter-organ interactions that are crucial for understanding complex diseases (Lee and Sung, 2018). For instance, gut–liver-on-chip platforms simulate enterohepatic circulation to examine the influence of changes in gut barrier function on liver lipid accumulation and inflammation (Velliou et al., 2024). Such integrated systems provide insight into disease pathogenesis and facilitate the study of systemic drug responses, thereby enhancing translational predictability (Soto et al., 2024). Furthermore, integrating liver-on-chip systems with adipose tissue or pancreatic modules has begun to elucidate the intricate endocrine and paracrine factors contributing to MASLD progression and insulin resistance (Morisseau, 2023).

An additional translational advantage lies in integrating artificial intelligence (AI) and machine learning tools with organ-on-chip data to analyze large-scale datasets and reveal subtle patterns in drug responses and disease progression (De Chiara et al., 2021). Such computational approaches facilitate the identification of novel biomarkers and enable real-time adjustments to experimental parameters, ultimately leading to more robust predictions of clinical outcomes (Rezvani et al., 2023).

Despite these significant advancements, there are still several challenges in developing and widely adopting liver-on-chip models (Polidoro et al., 2021). One of the primary obstacles is the technical complexity of fabricating and operating these microfluidic devices, especially with regard to integrating multiple cell types reproducibly (Messelmani et al., 2022). Another critical issue is maintaining the long-term viability and functionality of primary human hepatocytes within the chip, as traditional 2D cultures rapidly lose their hepatic phenotype and even 3D cultures can struggle with nutrient and oxygen diffusion in thicker tissues (Hassan et al., 2020). Although liver-on-chip models can accurately simulate certain aspects of the liver microenvironment, they still struggle to replicate the full complexity of human liver tissue, including the incorporation of immune components and the recreation of dynamic inter-organ interactions (Velliou et al., 2024; Rezvani et al., 2023). Despite the ongoing challenges related to scalability, standardization and long-term cell viability, technological advances and the integration of multi-organ systems and AI-based analytics are expected to overcome these barriers (Velliou et al., 2024).

## 5 *Ex-vivo* models

The principal of all *ex vivo* models is the isolation of a specific piece of human or animal organ, tissue or biopsy, which is subsequently maintained in a state of physiological activity. These models provide the original architecture of the tissue, including the composition of cells and extracellular matrix. The implementation of *ex vivo* models facilitates the effective realization of the 3R strategy. The use of human tissue samples replaces the necessity for animal experiments for some scientific questions, while the employment of animal organs enables the reduction and refinement of the number of animals required for study. It is crucial to obtain approval from the human research ethics committee before using human samples in *ex vivo* experiments. All participants must be informed about the aim of the study and must provide written informed consent prior to participation.

*Ex vivo* experiments with isolated human biopsies represent a crucial tool in the field of gastroenterology. For example, biopsies obtained from patients with IBD and irritable bowel syndrome (IBS) provide invaluable insights into the complexities of these disorders, which are challenging to replicate in animal models. Moreover, no animal models can perfectly recapitulate the clinical and pathophysiological alterations characteristic of IBD or IBS (Eichele and Kharbanda, 2017; Kiesler et al., 2015; Nasser et al., 2014; Pe et al., 2018). Isolated intestinal and liver tissue from animals can be used to obtain PCTS. As it is an ideal and reliable model for studying a range of diseases affecting the liver and digestive system and for evaluating the efficacy of new drugs.

### 5.1 Ussing chambers

The Ussing chamber technique was developed by the Danish scientist Hans H. Ussing. It is a robust and valuable electrophysiological method that enables the study of drug transport, the movement and permeability of ions, nanoparticles, and active substances across intestinal tissue in a laboratory setting. In addition, this method allows to investigate bacterial interactions with hosts (Pe et al., 2018; Larsen, 2002; Streekstra et al., 2022; Gardey et al., 2024; Clarke, 2009; Mendes et al., 2018). Furthermore, the FDA recommends the use of human intestinal tissue for permeability studies (United States. Department of Health and Human Services, issuing body et al., 2017). It has been demonstrated that the results obtained from animal experiments are not always aligned with those observed in clinical studies. Nevertheless, the number of studies using human mucosa remains relatively limited (Nunes et al., 2016; Gardey et al., 2024; Lautenschläge et al., 2013; Schmidt et al., 2013).

**FIGURE 2:**
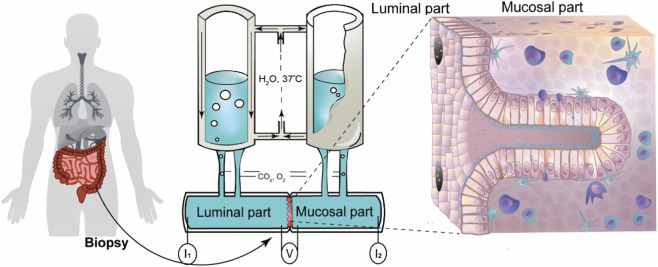
The scheme of Ussing chamber and the orientation of human mucosa in the chamber. V–voltage electrodes connected to the voltmeter, I–current electrodes connected to the current source. This simplified drawing of the colon mucosa shows the intestinal crypt, the epithelial barrier and the lamina propria, which contains immune cells (Gardey, 2022).

Figure 2 illustrates the schematic representation of the Ussing chamber and the orientation of human mucosa. An isolated tissue biopsy is mounted between two chamber compartments and filled with a physiological solution, typically Krebs bicarbonate Ringer (KBR) buffer. A circulating water jacket maintains the buffer temperature at 37 °C, which approximates the body temperature. The carbogen gas oxygenates and agitates the physiological solution within the Ussing chamber. Overall, the chamber is designed to extend the viability of the tissue outside the body. The viability of the tissue is controlled via electrophysiological parameters. The set of current and voltage electrodes are connected to the chamber via agar-salt bridges. Voltage/Current Clamp software monitors tissue resistance or its inverse transepithelial conductance, potential difference, voltage and tissue current (Clarke, 2009; Snelson et al., 2024; Gardey, 2022; Thomson et al., 2019; Stockmann et al., 1999; McNamara et al., 1999). When the entire organ is available for the experiment, the thickness of the tissue must be considered. Therefore, the seromusculature layers are typically removed before mounting tissue into the chamber, as additional layers may impede the transport of nutrients and oxygen. Furthermore, rhythmic neuromuscular contractions of the entire intestine could potentially induce alterations in the electrophysiological parameters of the tissue (Clarke, 2009). It is essential to handle the tissue with the highest degree of precaution in order to avoid any destruction or damage to the epithelium.

Epithelial tissue is characterized by the properties of polarity and tightness, which are fundamental to its structure and function (Li et al., 2004). Tight junctions play a critical role in maintaining the integrity of the mucosa, which subsequently impacts tissue resistance. To determine the resistance, bipolar current pulses are applied from the current source while monitoring the voltage using a voltmeter. In accordance with Ohm’s law, the resistance is equal to the voltage divided by the current. If the resistance is too low, the tissue is damaged. It is essential to verify the viability of tissue during the entire experiment. In order to achieve this, the short-circuit current (I_SC_) of the tissue is measured. The I_SC_ demonstrates the active flux of ions through the tissue. Following the disconnection of the current source, the voltages on the electrodes I_1_ and I_2_ are equal to zero (V = 0), and the voltmeter measures the potential difference (PD) created by the active transport of ions in the mucosa against an electrical- or concentration-based gradient, which reflects the tissue viability (Clarke, 2009; Gardey, 2022; Polentarutti et al., 1999; Sjögren et al., 2016; Westerhout et al., 2015). Furthermore, the viability of tissue can be evaluated through the monitoring of forskolin, theophylline, the transport inhibitor bumetanide, or prostaglandin E2, which alters ion transport and consequently affects I_SC_ within viable tissue. Moreover, the integrity of the tissue can be controlled by Lucifer yellow or 4 kDa FITC-dextran (Clarke, 2009; Thomson et al., 2019; Stockmann et al., 1999; McNamara et al., 1999).

Ussing chamber technique allows for the investigation of novel methods of drug delivery systems across gastrointestinal barriers, particularly in patients with IBD (Gardey et al., 2024). Moreover, *ex vivo* experiments with native tissue isolated from patients with celiac disease represents a crucial tool for evaluation of intestinal permeability in this gluten-related disorder (Biskou and Jauregi-Miguel, 2023). The principal advantage of *ex vivo* models is the opportunity to conduct experiments with functional, live tissue outside of the organism (Xu et al., 2021). Nevertheless, this model is limited by the complexity of the experimental setup and the loss of tissue viability during tissue handling (Nunes et al., 2016; Clarke, 2009). The viability of tissue in these *ex vivo* experiments (2–3 h) restricts the feasibility of certain experiments that require prolonged incubation. Moreover, the majority of research teams are unable to utilize freshly isolated biopsies, which limits the widespread practical application of this method (Table 4) (Gardey, 2022).

**TABLE 4:** Advantages and disadvantages of the Ussing chamber technique.

Advantages	Disadvantages
Suitable for human tissue	Limited tissue viability
Investigation of different intestinal regions	Complex experimental set up
Preserves physiological and morphological characteristics of the intestine	Fragile tissue requires careful handling
Applicable across multiple animal species (mouse, rat, pig)	Low throughput and labor-intensive
Allows parallel testing of several compounds on the same donor	Limited excess to freshly isolated tissue

### 5.2 Everted gut sac method

The everted gut sac technique is used to analyze absorption and permeability in the intestine (Table 5). This *ex vivo* method was first described in 1954 by Wilson and Wiseman who studied the absorption of glucose and methionine in rats and hamsters (Wilson and Wiseman, 1954). Since then, the everted gut sac model has evolved methodically and is now frequently used to investigate the kinetics and mechanisms of drug and nutrient absorption (Rahman et al., 2021; Alam et al., 2012). It is also utilized to assess intestinal toxicity of substances and to improve the absorption of pharmacological active compounds (Binkhathlan et al., 2024; Gu et al., 2018). This method is often applied in mice and rats but can be used in varying species including sheep, rabbits, hamsters, pigs, chickens and fish (Alam et al., 2012). In addition, results can be investigated mechanistically by using animals with a specific knockout and the impact of diseases such as sepsis or IBD on absorption and permeability in the intestine can also be analyzed (Oami and Coopersmith, 2021; Hayashi et al., 2023; Kazgan et al., 2014).

**TABLE 5:** Advantages and disadvantages of the everted gut sac model.

Advantages	Disadvantages
Intact animal tissue (Rahman et al., 2021)	Limited tissue availability (Rahman et al., 2021)
Relatively large surface area for absorption (Alam et al., 2012)	Not suitable for human intestine (Alam et al., 2012)
Investigation of different intestinal regions (Rahman et al., 2021)	Loss of enzymatic activity (Alam et al., 2012)
Presence of a mucus layer (Rahman et al., 2021)	Injury of the intestinal tissue while inverting (Hamilton and Butt, 2013)
Applicable across multiple animal species (mouse, rat, sheep, pig, broilers, fish) (Rahman et al., 2021; Sui et al., 2011; Kodzhahinchev et al., 2018)	Small closed serosal compartment influences uptake studies of rapidly absorbed substances (Gandia et al., 2011)
Fast and inexpensive (Barthe et al., 1998)	Presence of a seromuscular layer that must be crossed may lead to underestimation of absorption (Noorman et al., 2024)
Allows for parallel testing of several compounds (Álvarez-Olguín et al., 2022)	​
Small serosal volume allows for rapid increase in concentration (Wilson and Wiseman, 1954)	​

**FIGURE 3:**
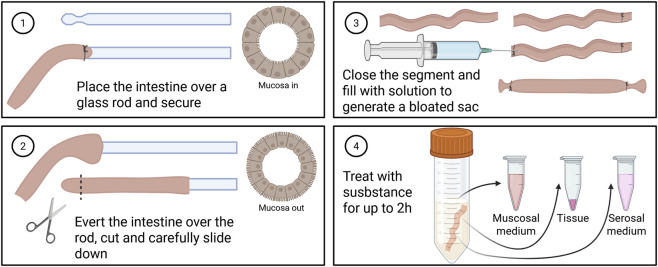
Schematic representation of the everted gut sac method. Created in BioRender. Lossow, K. (2026). https://BioRender.com/wxfc1jv, licensed under CC BY 4.0.

To generate the everted sac (Figure 3), the entire intestine is removed as soon as possible after the animal is sacrificed and the mesentery is removed without damaging the tissue (Hamilton and Butt, 2013; Barthe et al., 1998). The intestine is washed with Ringer´s solution and cut into segments of 3–6 cm, depending on the species and size of the intestine. The segments are attached to a glass rod with braided silk and everted in the rod to shift the mucosal side of the intestine to the outside. One end of the segment is closed with a suture and then filled with Ringer´s solution and closed with a second suture to create a slightly bloated sac. The sac is placed in an oxygenated Ringer´s solution containing the substance for analysis (Hamilton and Butt, 2013; Barthe et al., 1998). Under physiological conditions at 37 °C the tissue is metabolically active and intact for about 2 hours (Barthe et al., 1998; Gandia et al., 2011). After the incubation time, the sac is removed, washed and cut open over a small tube to collect the serosal fluid to use for measurement (Hamilton and Butt, 2013). The mucosal fluid as well as the tissue itself can also be used to analyze the substance of interest (Hamilton and Butt, 2013). It is also possible to modify this model by making a non-everted sac, which is filled with the substance to be analyzed and placed in an acceptor solution (Alam et al., 2012). Instead of absorption, the permeability of the intestine towards different sized macromolecules can also be determined e.g., with the help of FITC-labeled dextrans of different molecular weights: 4, 10, 70 and 150 kDa (Oami and Coopersmith, 2021; Gleeson et al., 2021).

The everted gut sac model depends on different factors like species, intestinal region, diet, disease state but also the animals age (Alam et al., 2012). Gleeson et al. showed that young mice express lower amounts of tight junctions in the small intestine compared to adult mice. This leads to a higher permeability and increased absorption via the paracellular route (Gleeson et al., 2021). There are also experimental factors such as incubation temperature, pH, concentration of substances and surface area that need to be considered when conducting experiments (Alam et al., 2012; Ruan et al., 2021). Findings from this model have been in line with *in vivo* investigations but compared to the Caco-2 cell monolayer the Caco-2 cells exhibit slightly smaller microvilli and show a lower paracellular transport (Barthe et al., 1998). It was also observed that there are enzymatic differences between the everted sac model and the Caco-2 cell monolayer since the sac´s have a higher brush border membrane peptidase-induced hydrolysis which can influence the absorption of small peptides (Wang et al., 2019). The everted gut sac model can be a useful tool to investigate absorption and toxicity under physiological and pathophysiological conditions.

### 5.3 Precision-cut liver and intestinal slices

**FIGURE 4:**
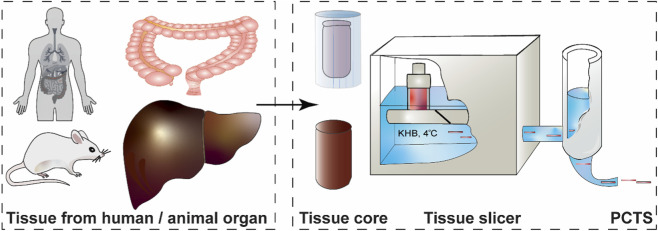
Schematic representation of precision cut liver and intestinal slices models. PCTS, precision cut tissue slices; KHB, Krebs-Henseleit buffer.

PCTS represent a powerful *ex vivo* tissue culture technique that provide the multicellular composition of the organ with intact intercellular and cell-matrix interactions and tissue homeostasis (Graaf et al., 2007; de Graaf et al., 2010; Majorova et al., 2021). In 1980, Krumdieck developed a device for cutting thin tissue slices. The ability to produce reproducible thin slices is invaluable for conducting a large number of prolonged *ex vivo* experiments using tissue from human or animal organs (Othman et al., 2020; Krumdieck et al., 1980). The prolonged viability of the slices represents a significant advantageous of PCTS method. Furthermore, the sufficient number of slices obtained from the organ provides the opportunity to implement a variety of conditions in the experiment (Majorova et al., 2021). Moreover, the results obtained from PCTS demonstrated a promising extrapolation to *in vivo* conditions (Olinga and Schuppan, 2013). Figure 4 represents the scheme of the model, which is described in further detail below. A comprehensive protocol for the preparation and incubation of PCTS was developed by de Graaf and colleagues (de Graaf et al., 2010).

While there is currently no roadmap for the standardization of PCTS, regarding the FDA Modernization Act 2.0, *ex vivo* human tissue is a key “New approach Methodology”. Therefore, there may be increased efforts to implement *ex vivo* tests in preclinical settings and to reduce animal experiments (Gerke et al., 2026).

#### 5.3.1 Intestine

As previously discussed, the Ussing chamber technique represents a crucial methodology in the field of gastroenterology. However, the viability of the biopsies is restricted to a timeframe of 2–3 h. In comparison, precision cut intestinal slices (PCIS) offer the potential to extend the tissue viability up to 24 h. Human tissue is typically obtained from patients following surgical procedures such as pancreatoduodenectomy, hemicolectomy, or clinically indicated surgeries (Pham et al., 2015; Hung et al., 2023; Li et al., 2018). The intestinal tissue has to be transferred in an ice-cold oxygenated Krebs-Henseleit buffer (KHB). The lumen must be flushed with the same buffer. In the case of human tissue, the muscularis must be removed from the mucosa and submucosa. The presence of a thick and taut muscularis in PCIS restricts the diffusion of nutrients and oxygen. In the case of a rat intestine, it is necessary to remove any adherent fatty tissue.

Due to the high sensitivity of the intestine to both warm and cold ischemia, the preparation process must be completed within a maximum of 2 hours. Intestinal tissue samples 10 mm × 20 mm should be embedded prior to cutting in agarose. Intestinal tissue is typically sliced into sections of approximately 250–300 µm in size and subsequently cultured in a supplemented William’s Medium E (de Graaf et al., 2010). Recently, it was shown that the tailor-made organoid media can enhance the viability of the PCIS for up to 72 h (Biel et al., 2022). PCIS has a number of potential applications in scientific research, including the investigation of drug metabolism, enzyme regulation, drug transport and intestinal toxicity, drug-drug interactions, antimicrobial drugs, and the screening of food allergens. In addition, it has been used in the analysis of intestinal fibrosis and related conditions (Pham et al., 2015; Hung et al., 2023; Niu et al., 2013; Groothuis et al., 2013; Iswandana et al., 2020; Martinec et al., 2021).

The viability of the PCTS can be monitor via the determination of ATP, water‐soluble tetrazolium salt (metabolic) assays, or lactate dehydrogenase assay and tissue morphology (de Graaf et al., 2010; Hung et al., 2023).

#### 5.3.2 Liver

Precision-cut liver slices (PCLS) retain liver structure, maintain zone-specific cytochrome activity and mechanisms of toxicity, demonstrate a perfect correlation for translational research (Lerche-Langrand and Toutain, 2000). The thickness of the tissue slices permits the diffusion of oxygen and nutrients, thereby extending the viability of the tissue. The viability of PCLS can be maintained for up to 5 days, with the potential for extension to 15 days under specific conditions (Palma et al., 2019; Wu et al., 2018). Liver samples for PCLS are typically obtained after partial hepatectomy, from surgical waste, or from non-transplantable tissue. The choice of whether to use a healthy organ or an organ affected by disease (fibrosis, cirrhosis or cancer) is ultimately determined by the research question and hypothesis. The study of immunological interactions is possible when PCLS are incubated with autologous mononuclear cells from peripheral blood. Additionally, livers from animals may be employed in this methodology. A large number of slices allows to support the concepts of reduction and refinement in accordance with the 3R principles.

Liver tissue samples must be flushed to remove blood with a University of Wisconsin solution. The tissue core can then be prepared using a cylindrical tissue drill to cut 1 cm cores with 5 mm diameters. Liver tissue can be cut directly in the slicer chamber. The use of a Krumdieck tissue slicer, a Brendel-Vitron slicer, or a Leica VT1200 Vibratome is recommended. The tissue cores must be cut at 4 °C in KHB gassed with carbogen on slices approximately 250 µm thick. This thickness represents approximately 10–15 cellular layers (Palma et al., 2019). PCLS are cultured in a supplemented version of Williams medium E in six- or 12-well plates. The medium must be refreshed every 24 h (de Graaf et al., 2010).

A variety of conditions can be investigated using PCLS, including alcohol-related and non-alcohol related steatohepatitis, as well as inflammation, fibrosis, cirrhosis, hepatocellular carcinoma, viral hepatitis, cholestatic disease, and drug-induced liver injury. Furthermore, the fibrogenic processes observed in rodents may not be representative of those occurring in humans. Therefore, the use of human PCLS allows for the investigation of human liver fibrosis (Olinga and Schuppan, 2013; Palma et al., 2019; Westra et al., 2016). PCLS are a commonly used tool in a number of scientific fields, including toxicological and pharmacological research, the study of liver diseases, drug delivery, and gene therapy (Dewyse et al., 2021). This *ex vivo* method is being employed to investigate a number of processes, including metabolic studies, toxicity, and fibrosis (Othman et al., 2020). In contrast to isolated cells, hepatocytes in PCLS do not exhibit loss of intracellular polarity (de Graaf et al., 2010). One of the limitations of this *ex vivo* method for investigating liver disease is the activation of hepatic stellate cells, which can lead to fibrotic reactions due to manipulation (Dewyse et al., 2021).

The most obvious limitation of this method is the difficulty of accessing the fresh liver and intestines. The good coordination between physicians, surgeons, nurses and researchers are required. While human tissue is an invaluable resource for toxicity studies and the investigation of liver diseases, the reproducibility of results significantly challenged by the considerable variability observed in patients and the quality of tissue, which is influenced by genetic and disease factors (Biel et al., 2022; Dewyse et al., 2021). Furthermore, the viability of PCTS is reliant on the quality of the organ, which is often compromised by disease (de Graaf et al., 2010). Nevertheless, PCTS represent a powerful tool for a personalized medicine. However, further validation and comparison with *in vivo* and *in vitro* experiments are necessary (Olinga and Schuppan, 2013).

## 6 Conclusion and future directions

Only 32% of drugs from preclinical phase move to clinical trial phase (Takebe et al., 2018). One of the primary reasons for the significant failure rate in the transition of novel drugs to the clinical phase is the insufficient representation of human physiology and pathophysiology by *in vitro* and *in vivo* models (Malaguarn et al., 2021; Gardey et al., 2025). Therefore, it is necessary to investigate, establish, and improve alternative models. In the present review, we discussed the currently available models that have been implemented in the fields of hepatology and gastroenterology. Future cell culture technologies aim to better replicate the *in vivo* liver environment through innovations such as 3D co-culture systems, organoids, and organ-on-chip platforms that integrate fluid flow, multiple cell types, and physiological gradients. These advanced models hold promise for improving the predictive power of *in vitro* toxicity testing while reducing animal use and enhancing translational relevance for human health.

### 6.1 Protocol: How to choose an appropriate model?

The suitability of alternative models depends on the research context. While 2D and spheroid systems remain valuable for high-throughput toxicity screening, organoids and organ-on-chip platforms offer superior relevance for disease modeling and personalized medicine due to their enhanced physiological complexity. Integrated multi-tissue systems further improve predictive performance in pharmacokinetic and pathophysiological studies, highlighting a shift toward application-tailored model selection.

While animal tests in the cosmetic area are forbidden in Europe, the current trend is the general reduction of animal experiments, including tests in pharmaceutical and healthcare research and industries. Thus, alternative non-animal approaches are constantly developing and need further investigation (Parlament, 2021; Richter, 2024).

The following questions must be addressed and considered during the planning phase of the experiment:

It is necessary to determine whether the animal model is required.
 - The use of animal experimentation should be replaced wherever feasible.
- The number of animals used in experiments should be minimized where feasible. One approach to doing so would be to employ a mini-experiment design with interim analyses, thereby reducing the overall number of animals utilized in the experiment.
Explore the alternative models.
Justify the scientific quality of the alternative model.
Reliability of alternative model compared with animal model.
Is it a study of cell-cell interactions?
Is it a study of organ-organ interactions?
The costs of the model.

**FIGURE 5:**
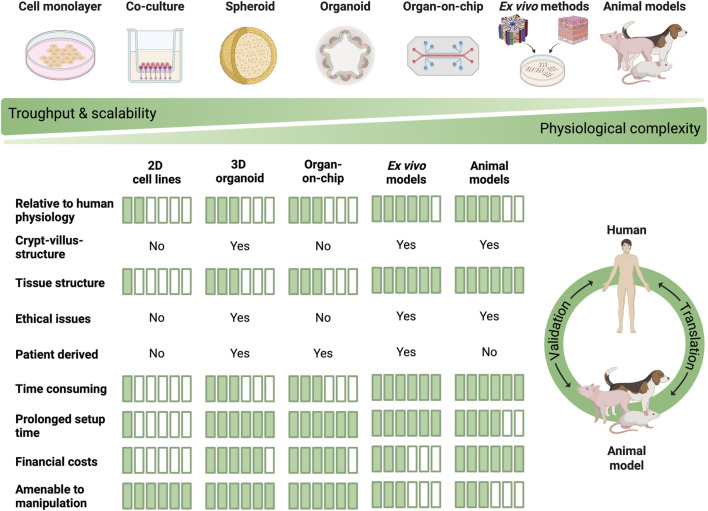
Schematic comparison of alternative models in gastrointestinal and hepatic studies. Created in BioRender. Börmel L. (2026) https://BioRender.com/bv234f5, licensed under CC BY 4.0.

It is obvious that each model has its own set of advantages and disadvantages (see Figure 5). Therefore, it is essential to select an alternative model with a clear understanding of the research question and scientific hypothesis. Additionally, it is important to address the reproducibility aspect of the alternative models. The main sources of variability include the origin of the cell sources, donor and patient heterogeneity, differences in disease stages, additional health complications in patient-derived models, and the lack of harmonized and standardized protocols. At the same time, increasing efforts are being made to establish the guidelines and initiate discussions between academical and institutional laboratory to address these challenges.

### 6.2 Future directions

Most probably, the machine learning methods currently being developed will become an essential source of information. This will help to reduce the number of animal experiments. The machine learning will provide important predictive information about drug-drug interactions, toxicity, absorption, etc (Gheorghita et al., 2025). Even in the future, it is clear that we cannot skip *in vitro*, *ex vivo* and *in vivo* experiments. However, new machine learning methods will help reduce the time, effort and cost of these experiments.

To select the most suitable methods as an alternative to animal experiments, it is important to consider human physiology and pathophysiology. Then, the most effective technique can be selected for testing the hypothesis, bearing in mind the limitations and challenges of each model. In general, researchers tend to start with the simplest model and move towards the most complex one. However, the closer the model is to human physiology, the more translatable the results will be. Currently, the majority of results obtained from *in vitro* experiments cannot be reproduced *in vivo* or in clinical settings. A better understanding of *in vitro* experiments and the development and implementation of more physiologically relevant models can help to advance basic science to the clinical stage.

In conclusion, the implementation of standardized and robust alternatives to animal models would be more cost-effective and time-saving. However, it is unlikely that animal models will be completely replaced in the research and pharmaceutical industries in the near future. The successful integration of alternative methods into drug development, for example, requires dedicated collaboration between academic institutes, clinicians, regulatory authority, and industry.

### 6.3 Limitations

As a narrative review, this work does not present a systematic literature search. Instead, it reflects an expert synthesis of current knowledge, informed by the authors’ research experience and engagement within the field. Readers should consider the inherent limitations of such an approach, including potential selection bias.

Throughout the review, we emphasize applications in MASLD and IBD, as these conditions represent examples of complex, multifactorial diseases in hepatology and gastroenterology. MASLD involves a dynamic interplay of metabolic, inflammatory, and fibrotic processes that are only partly recapitulated in rodents, while IBD requires modeling of epithelial barrier function, host-microbe interactions, and immune crosstalk. These features are increasingly addressed by advanced human-based models such as organoids and gut-on-chip systems. By highlighting these areas, we aim to illustrate how next-generation *in vitro* platforms can address unmet needs in disease modeling and drug development. However, the advantages and findings discussed for these disease areas cannot be automatically generalized to other hepatic or intestinal disorders. MASLD and IBD represent specific pathophysiological contexts, and the performance and translational relevance of advanced human-based *in vitro* models may differ across disease entities. Therefore, disease-specific validation remains essential when applying these platforms to other diseases or a healthy environment.

## References

[B1] Abuwatfa W. H. Pitt W. G. Husseini G. A. (2024). Scaffold-based 3D cell culture models in cancer research. J. Biomed. Sci. 31, 7. 10.1186/s12929-024-00994-y 38221607 PMC10789053

[B2] Adams D. H. Ju C. Ramaiah S. K. Uetrecht J. Jaeschke H. (2010). Mechanisms of immune-mediated liver injury. Toxicol. Sci. 115, 307–321. 10.1093/toxsci/kfq009 20071422 PMC2871750

[B3] Ahn S.-J. Lee S. Kwon D. Oh S. Park C. Jeon S. (2024). Essential guidelines for manufacturing and application of organoids. Int. J. Stem Cells 17, 102–112. 10.15283/ijsc24047 38764240 PMC11170116

[B4] Ahn S.-J. (2024). Standards for organoids. Int. J. Stem Cells 17, 99–101. 10.15283/ijsc24043 38798276 PMC11170121

[B5] Alam M. A. Al-Jenoobi F. I. Al-Mohizea A. M. (2012). Everted gut sac model as a tool in pharmaceutical research: limitations and applications. J. Pharm. Pharmacol. 64, 326–336. 10.1111/j.2042-7158.2011.01391.x 22309264

[B6] Almeqdadi M. Mana M. D. Roper J. Yilmaz Ö. H. (2019). Gut organoids: mini-tissues in culture to study intestinal physiology and disease. Am. J. Physiol. Cell Physiol. 317, C405–C419. 10.1152/ajpcell.00300.2017 31216420 PMC6766612

[B7] Alonso-Roman R. Mosig A. S. Figge M. T. Papenfort K. Eggeling C. Schacher F. H. (2024). Organ-on-chip models for infectious disease research. Nat. Microbiol. 9, 891–904. 10.1038/s41564-024-01645-6 38528150

[B8] Álvarez-Olguín M. A. Beltrán-Barrientos L. M. Hernandez-Mendoza A. González-Córdova A. F. Vallejo-Cordoba B. (2022). Current trends and perspectives on bioaccessibility and bioavailability of food bioactive peptides: *in vitro* and *ex vivo* studies. J. Sci. Food Agric. 102, 6824–6834. 10.1002/jsfa.12077 35716022

[B9] American Type Culture Collection (ATCC) (2025). McA-RH7777 (CRL-1601) product sheet.

[B10] Andrews M. G. Kriegstein A. R. (2022). Challenges of organoid research. Annu. Rev. Neurosci. 45, 23–39. 10.1146/annurev-neuro-111020-090812 34985918 PMC10559943

[B11] Angus H. C. K. Butt A. G. Schultz M. Kemp R. A. (2019). Intestinal organoids as a tool for inflammatory bowel disease research. Front. Med. (Lausanne) 6, 334. 10.3389/fmed.2019.00334 32010704 PMC6978713

[B12] Anthérieu S. Chesné C. Li R. Guguen-Guillouzo C. Guillouzo A. (2012). Optimization of the HepaRG cell model for drug metabolism and toxicity studies. Toxicol. Vitro 26, 1278–1285. 10.1016/j.tiv.2012.05.008 22643240

[B13] Arzumanian V. A. Kiseleva O. I. Poverennaya E. V. (2021). The curious case of the HepG2 cell line: 40 years of expertise. IJMS 22, 13135. 10.3390/ijms222313135 34884942 PMC8658661

[B14] Ashkenas J. Muschler J. Bissell M. J. (1996). The extracellular matrix in epithelial biology: shared molecules and common themes in distant phyla. Dev. Biol. 180, 433–444. 10.1006/dbio.1996.0317 8954716 PMC3815653

[B15] ATCC (2025). American type culture collection (ATCC). AML12 (CRL-2254) product sheet.

[B17] Banavar S. R. Rawal S. Y. Pulikkotil S. J. Daood U. Paterson I. C. Davamani F. A. (2021). 3D clumps/extracellular matrix complexes of periodontal ligament stem cells ameliorate the attenuating effects of LPS on proliferation and osteogenic potential, J. Pers. Med. 11, 528 10.3390/jpm11060528 34207600 PMC8227185

[B18] Banerjee P. Senapati S. (2024). Translational utility of organoid models for biomedical research on gastrointestinal diseases. Stem Cell Rev. Rep. 20, 1441–1458. 10.1007/s12015-024-10733-3 38758462

[B19] Bao L. Cui X. Bai R. Chen C. (2023). Advancing intestinal organoid technology to decipher nano-intestine interactions and treat intestinal disease. Nano Res. 16, 3976–3990. 10.1007/s12274-022-5150-4 36465523 PMC9685037

[B20] Baratta J. L. Ngo A. Lopez B. Kasabwalla N. Longmuir K. J. Robertson R. T. (2009). Cellular organization of normal mouse liver: a histological, quantitative immunocytochemical, and fine structural analysis. Histochem Cell Biol. 131, 713–726. 10.1007/s00418-009-0577-1 19255771 PMC2761764

[B21] Bar-Ephraim Y. E. Kretzschmar K. Clevers H. (2020). Organoids in immunological research. Nat. Rev. Immunol. 20, 279–293. 10.1038/s41577-019-0248-y 31853049

[B22] Barker N. Huch M. Kujala P. van de Wetering M. Snippert H. J. van Es J. H. (2010). Lgr5(+ve) stem cells drive self-renewal in the stomach and build long-lived gastric units *in vitro* . Cell Stem Cell 6, 25–36. 10.1016/j.stem.2009.11.013 20085740

[B23] Barthe L. Woodley J. F. Kenworthy S. Houin G. (1998). An improved everted gut sac as a simple and accurate technique to measure paracellular transport across the small intestine. Eur. J. Drug Metab. Pharmacokinet. 23, 313–323. 10.1007/BF03189357 9725499

[B24] Baudy A. R. Otieno M. A. Hewitt P. Gan J. Roth A. Keller D. (2020). Liver microphysiological systems development guidelines for safety risk assessment in the pharmaceutical industry. Lab. Chip 20, 215–225. 10.1039/c9lc00768g 31799979

[B25] Baumert B. Maretti-Mira A. C. Walker D. I. Li Z. Stratakis N. Wang H. (2025). Integrated spheroid-to-population framework for evaluating PFHpA-Associated metabolic dysfunction and steatotic liver disease. Preprint. 10.21203/rs.3.rs-5960979/v1 40092438 PMC11908348

[B26] Beamer M. A. Zamora C. Nestor-Kalinoski A. L. Fernando V. Sharma V. Furuta S. (2023). Novel 3D flipwell system that models gut mucosal microenvironment for studying interactions between gut microbiota, epithelia and immunity. Sci. Rep-Uk 13, 870. 10.1038/s41598-023-28233-8 36650266 PMC9845379

[B27] Bein A. Shin W. Jalili-Firoozinezhad S. Park M. H. Sontheimer-Phelps A. Tovaglieri A. (2018). Microfluidic Organ-on-a-Chip models of human intestine. Cell. Mol. Gastroenterol. Hepatol. 5, 659–668. 10.1016/j.jcmgh.2017.12.010 29713674 PMC5924739

[B28] Beterams A. De Paepe K. Maes L. Wise I. J. De Keersmaecker H. Rajkovic A. (2021). Versatile human *in vitro* triple coculture model coincubated with adhered gut microbes reproducibly mimics pro-inflammatory host-microbe interactions in the Colon. FASEB J. 35, e21992. 10.1096/fj.202101135R 34719821

[B29] Betge J. Rindtorff N. Sauer J. Rauscher B. Dingert C. Gaitantzi H. (2022). The drug-induced phenotypic landscape of colorectal cancer organoids. Nat. Commun. 13, 3135. 10.1038/s41467-022-30722-9 35668108 PMC9170716

[B30] Bhatia S. N. Ingber D. E. (2014). Microfluidic organs-on-chips. Nat. Biotechnol. 32, 760–772. 10.1038/nbt.2989 25093883

[B31] Bianchi J. R. D. O. Carvalho B. G. Carvalho H. F. De La Torre L. G. (2025). Microfluidic-based gelatin methacrylate microgel as a scaffold to create reverse-polarity HT29 spheroids. Int. J. Biol. Macromol. 305, 140824. 10.1016/j.ijbiomac.2025.140824 39954894

[B32] Biel C. Martinec O. Sibering B. van Summeren K. Wessels A. M. A. Touw D. J. (2022). Extending the viability of human precision-cut intestinal slice model for drug metabolism studies. Archives Toxicol. 96, 1815–1827. 10.1007/s00204-022-03295-1 35428896 PMC9095520

[B33] Biganzoli E. Cavenaghi L. A. Rossi R. Brunati M. C. Nolli M. L. (1999). Use of a Caco-2 cell culture model for the characterization of intestinal absorption of antibiotics. Farmaco 54, 594–599. 10.1016/s0014-827x(99)00069-5 10555261

[B34] Binkhathlan Z. Ali R. Yusuf O. Alomrani A. H. Badran M. M. Alshememry A. K. (2024). Polycaprolactone-vitamin E TPGS micellar formulation for oral delivery of paclitaxel. Polym. (Basel) 16, 2232. 10.3390/polym16152232 39125257 PMC11314731

[B35] Bisconti F. Abreu H. Nguyen T. D. Stolfi F. Corbezzolo N. Gigli G. (2025). My cells, my model: immune-competent autologous organ-on-chip systems as a new paradigm in precision medicine. Front. Immunol. 16, 1712796. 10.3389/fimmu.2025.1712796 41459505 PMC12740941

[B36] Biskou O. Jauregi-Miguel A. (2023). “Measuring intestinal permeability in celiac disease *ex vivo*, using ussing chambers,”Methods Cell Biol. 179 21–38. 10.1016/bs.mcb.2022.12.005 37625877

[B37] Blouin A. Bolender R. P. Weibel E. R. (1977). Distribution of organelles and membranes between hepatocytes and nonhepatocytes in the rat liver parenchyma. A stereological study. J. Cell Biol. 72, 441–455. 10.1083/jcb.72.2.441 833203 PMC2110997

[B38] Bonanini F. Kurek D. Previdi S. Nicolas A. Hendriks D. de Ruiter S. (2022). *In vitro* grafting of hepatic spheroids and organoids on a microfluidic vascular bed. Angiogenesis 25, 455–470. 10.1007/s10456-022-09842-9 35704148 PMC9519670

[B39] Borah A. Kumar D. S. (2022). “Overcoming the barriers of two-dimensional cell culture systems with three-dimensional cell culture systems: techniques, drug discovery, and biomedical applications,” in Biomedical product and materials evaluation (Elsevier), 179–229. 10.1016/B978-0-12-823966-7.00003-7

[B40] Boran T. Zengin O. S. Seker Z. Gunaydin Akyildiz A. Oztas E. Özhan G. (2024). The cyclin-dependent kinase inhibitor abemaciclib-induced hepatotoxicity: insight on the molecular mechanisms in HepG2/THP-1 co-culture model. Toxicol. Lett. 391, 1–12. 10.1016/j.toxlet.2023.11.005 37992977

[B41] Cacciamali A. Villa R. Dotti S. (2022). 3D cell cultures: evolution of an ancient tool for new applications. Front. Physiol. 13, 836480. 10.3389/fphys.2022.836480 35936888 PMC9353320

[B42] Cao X. Gibbs S. T. Fang L. Miller H. A. Landowski C. P. Shin H. C. (2006). Why is it challenging to predict intestinal drug absorption and oral bioavailability in human using rat model. Pharm. Res. 23, 1675–1686. 10.1007/s11095-006-9041-2 16841194

[B43] Caon E. Martins M. Hodgetts H. Blanken L. Vilia M. G. Levi A. (2024). Exploring the impact of the PNPLA3 I148M variant on primary human hepatic stellate cells using 3D extracellular matrix models. J. Hepatol. 80, 941–956. 10.1016/j.jhep.2024.01.032 38365182

[B44] Cardoso B. D. Castanheira E. M. S. Lanceros‐Méndez S. Cardoso V. F. (2023). Recent advances on cell culture platforms for *in vitro* drug screening and cell therapies: from conventional to microfluidic strategies. Adv. Healthc. Mater. 12, 2202936. 10.1002/adhm.202202936 36898671 PMC11468737

[B45] Cerchiari A. Garbe J. C. Todhunter M. E. Jee N. Y. Pinney J. R. LaBarge M. A. (2015). Formation of spatially and geometrically controlled three-dimensional tissues in soft gels by sacrificial micromolding. Tissue Eng. Part C. Methods 21, 541–547. 10.1089/ten.TEC.2014.0450 25351430 PMC4442595

[B46] Cerec V. Glaise D. Garnier D. Morosan S. Turlin B. Drenou B. (2007). Transdifferentiation of hepatocyte-like cells from the human hepatoma HepaRG cell line through bipotent progenitor. Hepatol. 45, 957–967. 10.1002/hep.21536 17393521

[B47] Choi W. H. Bae D. H. Yoo J. (2023). Current status and prospects of organoid-based regenerative medicine. BMB Rep. 56, 10–14. 10.5483/BMBRep.2022-0195 36523211 PMC9887105

[B48] Clarke L. L. (2009). A guide to ussing chamber studies of mouse intestine. Am. J. Physiol. Gastrointest. Liver Physiol. 296, G1151–G1166. 10.1152/ajpgi.90649.2008 19342508 PMC2697950

[B49] Clement B. Guguen-Guillouzo C. Campion J. P. Glaise D. Bourel M. Guillouzo A. (1984). Long-term Co-Cultures of adult human hepatocytes with rat liver epithelial cells: modulation of albumin secretion and accumulation of extracellular material. Hepatol. 4, 373–380. 10.1002/hep.1840040305 6373549

[B50] Co J. Y. Margalef-Català M. Li X. Mah A. T. Kuo C. J. Monack D. M. (2019). Controlling epithelial polarity: a human enteroid model for host-pathogen interactions. Cell Rep. 26, 2509–2520.e4. 10.1016/j.celrep.2019.01.108 30811997 PMC6391775

[B51] Co J. Y. Margalef-Català M. Monack D. M. Amieva M. R. (2021). Controlling the polarity of human gastrointestinal organoids to investigate epithelial biology and infectious diseases. Nat. Protoc. 16, 5171–5192. 10.1038/s41596-021-00607-0 34663962 PMC8841224

[B52] Corsini N. S. Knoblich J. A. (2022). Human organoids: new strategies and methods for analyzing human development and disease. Cell 185, 2756–2769. 10.1016/j.cell.2022.06.051 35868278

[B53] Costello C. M. Sorna R. M. Goh Y. L. Cengic I. Jain N. K. March J. C. (2014). 3-D intestinal scaffolds for evaluating the therapeutic potential of probiotics. Mol. Pharm. 11, 2030–2039. 10.1021/mp5001422 24798584 PMC4096232

[B54] Cui X.-S. Li H. Z. Li L. Xie C. Z. Gao J. M. Chen Y. Y. (2025). Rodent model of metabolic dysfunction-associated fatty liver disease: a systematic review. J. Gastroenterol. Hepatol. 40, 48–66. 10.1111/jgh.16749 39322221 PMC11771679

[B55] Cushing M. C. Anseth K. S. (2007). Hydrogel cell cultures. Science 316, 1133–1134. 10.1126/science.1140171 17525324

[B56] D’Angelo E. Natarajan D. Sensi F. Ajayi O. Fassan M. Mammano E. (2020). Patient-derived scaffolds of colorectal cancer metastases as an organotypic 3D model of the liver metastatic microenvironment. Cancers 12, 364. 10.3390/cancers12020364 32033473 PMC7072130

[B57] Darling N. J. Mobbs C. L. González-Hau A. L. Freer M. Przyborski S. (2020). Bioengineering novel *in vitro* Co-culture models that represent the human intestinal mucosa with improved Caco-2 structure and barrier function. Front. Bioeng. Biotechnol. 8, 992. 10.3389/fbioe.2020.00992 32984279 PMC7487342

[B58] De Chiara F. Ferret-Miñana A. Ramón-Azcón J. (2021). The synergy between Organ-on-a-Chip and artificial intelligence for the study of NAFLD: from basic science to clinical research. Biomedicines 9, 248. 10.3390/biomedicines9030248 33801289 PMC7999375

[B59] de Graaf I. A. Olinga P. de Jager M. H. Merema M. T. de Kanter R. van de Kerkhof E. G. (2010). Preparation and incubation of precision-cut liver and intestinal slices for application in drug metabolism and toxicity studies. Nat. Protoc. 5, 1540–1551. 10.1038/nprot.2010.111 20725069

[B60] de Haan P. Santbergen M. J. C. van der Zande M. Bouwmeester H. Nielen M. W. F. Verpoorte E. (2021). A versatile, compartmentalised gut-on-a-chip system for pharmacological and toxicological analyses. Sci. Rep-Uk 11, 4920. 10.1038/s41598-021-84187-9 33649376 PMC7921645

[B61] Delon L. C. Guo Z. Oszmiana A. Chien C. C. Gibson R. Prestidge C. (2019). A systematic investigation of the effect of the fluid shear stress on Caco-2 cells towards the optimization of epithelial organ-on-chip models. Biomaterials 225, 119521. 10.1016/j.biomaterials.2019.119521 31600674

[B62] Devriese S. Van den Bossche L. Van Welden S. Holvoet T. Pinheiro I. Hindryckx P. (2017). T84 monolayers are superior to Caco-2 as a model system of colonocytes. Histochem Cell Biol. 148, 85–93. 10.1007/s00418-017-1539-7 28265783

[B63] Dewyse L. Reynaert H. van Grunsven L. A. (2021). Best practices and progress in precision-cut liver slice cultures. Int. J. Mol. Sci. 22, 7137. 10.3390/ijms22137137 34281187 PMC8267882

[B64] Ding X. Hu X. Chen Y. Xie J. Ying M. Wang Y. (2021). Differentiated Caco-2 cell models in food-intestine interaction study: current applications and future trends. Trends Food Sci. and Technol. 107, 455–465. 10.1016/j.tifs.2020.11.015

[B65] Donato M. T. Tolosa L. Gómez-Lechón M. J. (2015). Culture and functional characterization of human hepatoma HepG2 cells. Methods Mol. Biol. 1250, 77–93. 10.1007/978-1-4939-2074-7_5 26272135

[B66] Drolia R. Tenguria S. Durkes A. C. Turner J. R. Bhunia A. K. (2018). Listeria adhesion protein induces intestinal epithelial barrier dysfunction for bacterial translocation. Cell Host Microbe 23, 470–484.e7. 10.1016/j.chom.2018.03.004 29606495 PMC6750208

[B67] Dutton J. S. Hinman S. S. Kim R. Wang Y. Allbritton N. L. (2019). Primary cell-derived intestinal models: recapitulating physiology. Trends Biotechnol. 37, 744–760. 10.1016/j.tibtech.2018.12.001 30591184 PMC6571163

[B68] Eichele D. D. Kharbanda K. K. (2017). Dextran sodium sulfate colitis murine model: an indispensable tool for advancing our understanding of inflammatory bowel diseases pathogenesis. World J. Gastroenterol. 23, 6016–6029. 10.3748/wjg.v23.i33.6016 28970718 PMC5597494

[B69] Fedi A. Vitale C. Ponschin G. Ayehunie S. Fato M. Scaglione S. (2021). *In vitro* models replicating the human intestinal epithelium for absorption and metabolism studies: a systematic review. J. Control Release 335, 247–268. 10.1016/j.jconrel.2021.05.028 34033859

[B70] Feile A. Wegner V. D. Raasch M. Mosig A. S. (2024). Immunocompetent intestine-on-chip model for analyzing gut mucosal immune responses. J. Vis. Exp. 207. 10.3791/66603 38856194

[B71] Finkbeiner S. R. Freeman J. J. Wieck M. M. El-Nachef W. Altheim C. H. Tsai Y. H. (2015). Generation of tissue-engineered small intestine using embryonic stem cell-derived human intestinal organoids. Biol. Open 4, 1462–1472. 10.1242/bio.013235 26459240 PMC4728347

[B72] Fitzgerald K. A. Malhotra M. Curtin C. M. O’ Brien F. J. O’ Driscoll C. M. (2015). Life in 3D is never flat: 3D models to optimise drug delivery. J. Control. Release 215, 39–54. 10.1016/j.jconrel.2015.07.020 26220617

[B73] Fordham R. P. Yui S. Hannan N. R. F. Soendergaard C. Madgwick A. Schweiger P. J. (2013). Transplantation of expanded fetal intestinal progenitors contributes to Colon regeneration after injury. Cell Stem Cell 13, 734–744. 10.1016/j.stem.2013.09.015 24139758 PMC3858813

[B74] Gandia P. Lacombe O. Woodley J. Houin G. (2011). The perfused everted intestinal segment of rat. Arzneimittelforschung 54, 467–473. 10.1055/s-0031-1297000 15460214

[B75] Gardey E. Cseresnyes Z. Sobotta F. H. Eberhardt J. Haziri D. Grunert P. C. (2024). Selective uptake into inflamed human intestinal tissue and immune cell targeting by wormlike polymer micelles. Small 20, e2306482. 10.1002/smll.202306482 38109123

[B76] Gardey E. Brendel J. C. Stallmach A. (2025). Pathophysiology of IBD as a key strategy for polymeric nanoparticle development. Adv. Ther. 8, 2400439. 10.1002/adtp.202400439

[B77] Gardey E. (2022). Translocation of polymeric nanoparticles through the gastrointestinal barrier and their uptake by immune cells in inflammatory bowel disease. Jena, Germany: Friedrich-Schiller-Universität Jena.

[B78] Ge J.-Y. Wang Y. Li Q. L. Liu F. K. Lei Q. K. Zheng Y. W. (2024). Trends and challenges in organoid modeling and expansion with pluripotent stem cells and somatic tissue. PeerJ 12, e18422. 10.7717/peerj.18422 39619184 PMC11608026

[B79] Gerke S. Balamut J. Wagner J. K. (2026). The FDA’s plan to phase out animal testing. Trends Biotechnol., S0167779925005323. 10.1016/j.tibtech.2025.12.011 41571550 PMC12834477

[B80] Gheorghita F.-I. Bocanet V.-I. Iantovics L. B. (2025). Machine learning-based drug-drug interaction prediction: a critical review of models, limitations, and data challenges. Front. Pharmacol. 16, 1632775. 10.3389/fphar.2025.1632775 40808680 PMC12344460

[B81] Gleeson J. P. Fein K. C. Chaudhary N. Doerfler R. Newby A. N. Whitehead K. A. (2021). The enhanced intestinal permeability of infant mice enables oral protein and macromolecular absorption without delivery technology. Int. J. Pharm. 593, 120120. 10.1016/j.ijpharm.2020.120120 33249250 PMC7790917

[B82] Goers L. Freemont P. Polizzi K. M. (2014). Co-culture systems and technologies: taking synthetic biology to the next level. J. R. Soc. Interface. 11, 20140065. 10.1098/rsif.2014.0065 24829281 PMC4032528

[B83] Gómez-Lechón M. J. Jover R. Donato T. Ponsoda X. Rodriguez C. Stenzel K. G. (1998). Long-term expression of differentiated functions in hepatocytes cultured in three-dimensional collagen matrix. J. Cell Physiol. 177, 553–562. 10.1002/(SICI)1097-4652(199812)177:4<553::AID-JCP6>3.0.CO;2-F 10092208

[B84] Graaf I. A. M. de Groothuis G. M. M. Olinga P. (2007). Precision-cut tissue slices as a tool to predict metabolism of novel drugs. Expert Opin. Drug Metabolism and Toxicol. 3, 879–898. 10.1517/17425255.3.6.879 18028031

[B85] Granitzny A. Knebel J. Müller M. Braun A. Steinberg P. Dasenbrock C. (2017). Evaluation of a human *in vitro* hepatocyte-NPC co-culture model for the prediction of idiosyncratic drug-induced liver injury: a pilot study. Toxicol. Rep. 4, 89–103. 10.1016/j.toxrep.2017.02.001 28959630 PMC5615103

[B86] Groothuis G. M. M. de Graaf I. A. M. (2013). Precision-cut intestinal slices as *in vitro* tool for studies on drug metabolism. Curr. Drug Metab. 14, 112–119. 22497569

[B87] Grosheva I. Zheng D. Levy M. Polansky O. Lichtenstein A. Golani O. (2020). High-throughput screen identifies host and microbiota regulators of intestinal barrier function. Gastroenterology 159, 1807–1823. 10.1053/j.gastro.2020.07.003 32653496

[B88] Gu T. Yao C. Zhang K. Li C. Ding L. Huang Y. (2018). Toxic effects of zinc oxide nanoparticles combined with vitamin C and casein phosphopeptides on gastric epithelium cells and the intestinal absorption of mice. RSC Adv. 8, 26078–26088. 10.1039/c8ra03693d 35541949 PMC9082813

[B89] Guo L. Dial S. Shi L. Branham W. Liu J. Fang J. L. (2011). Similarities and differences in the expression of drug-metabolizing enzymes between human hepatic cell lines and primary human hepatocytes. Drug Metab. Dispos. 39, 528–538. 10.1124/dmd.110.035873 21149542 PMC3061558

[B90] Haag M. Winter S. Kemas A. M. Tevini J. Feldman A. Eder S. K. (2025). Circulating metabolite signatures indicate differential gut-liver crosstalk in lean and Obese MASLD. JCI Insight 10, e180943. 10.1172/jci.insight.180943 40100312 PMC12016937

[B91] Hahn U. Stallmach A. Hahn E. G. Riecken E. O. (1990). Basement membrane components are potent promoters of rat intestinal epithelial cell differentiation *in vitro* . Gastroenterology 98, 322–335. 10.1016/0016-5085(90)90821-h 2295387

[B92] Hamilton K. L. Butt A. G. (2013). Glucose transport into everted sacs of the small intestine of mice. Adv. Physiol. Educ. 37, 415–426. 10.1152/advan.00017.2013 24292921

[B93] Han D. W. Xu K. Jin Z. L. Xu Y. N. Li Y. H. Wang L. (2023). Customized liver organoids as an advanced *in vitro* modeling and drug discovery platform for non-alcoholic fatty liver diseases. Int. J. Biol. Sci. 19, 3595–3613. 10.7150/ijbs.85145 37497008 PMC10367556

[B94] Harrison S. P. Baumgarten S. F. Verma R. Lunov O. Dejneka A. Sullivan G. J. (2021). Liver organoids: recent developments, limitations and potential. Front. Med. 8, 574047. 10.3389/fmed.2021.574047 34026769 PMC8131532

[B95] Hassan S. Sebastian S. Maharjan S. Lesha A. Carpenter A. M. Liu X. (2020). Liver‐on‐a‐Chip models of fatty liver disease. Hepatology 71, 733–740. 10.1002/hep.31106 31909504 PMC7012755

[B96] Hayashi A. Sakamoto N. Kobayashi K. Murata T. (2023). Enhancement of prostaglandin D2-D prostanoid 1 signaling reduces intestinal permeability by stimulating mucus secretion. Front. Immunol. 14, 1276852. 10.3389/fimmu.2023.1276852 37942331 PMC10628818

[B97] Henry O. Y. F. Villenave R. Cronce M. J. Leineweber W. D. Benz M. A. Ingber D. E. (2017). Organs-on-chips with integrated electrodes for trans-epithelial electrical resistance (TEER) measurements of human epithelial barrier function. Lab a Chip 17, 2264–2271. 10.1039/c7lc00155j 28598479 PMC5526048

[B98] Higashi T. Friedman S. L. Hoshida Y. (2017). Hepatic stellate cells as key target in liver fibrosis. Adv. Drug Deliv. Rev. 121, 27–42. 10.1016/j.addr.2017.05.007 28506744 PMC5682243

[B99] Hilgendorf C. Ahlin G. Seithel A. Artursson P. Ungell A. L. Karlsson J. (2007). Expression of thirty-six drug transporter genes in human intestine, liver, kidney, and organotypic cell lines. Drug Metab. Dispos. 35, 1333–1340. 10.1124/dmd.107.014902 17496207

[B100] Ho S. M. Lewis J. D. Mayer E. A. Plevy S. E. Chuang E. Rappaport S. M. (2019). Challenges in IBD research: environmental triggers. Inflamm. Bowel Dis. 25, S13–s23. 10.1093/ibd/izz076 31095702 PMC6787673

[B101] Hughes C. S. Postovit L. M. Lajoie G. A. (2010a). Matrigel: a complex protein mixture required for optimal growth of cell culture. Proteomics 10, 1886–1890. 10.1002/pmic.200900758 20162561

[B102] Hughes C. S. Postovit L. M. Lajoie G. A. M. (2010b). A complex protein mixture required for optimal growth of cell culture. Proteomics 10, 1886–1890. 10.1002/pmgc.200900758 20162561

[B103] Hung L. Celik A. Yin X. Yu K. Berenjy A. Kothari A. (2023). Precision cut intestinal slices, a novel model of acute food allergic reactions. Allergy 78, 500–511. 10.1111/all.15579 36377289 PMC10098956

[B104] Ingber D. E. (2026). Challenges and opportunities for human organ chips in FDA assessments and pharma pipelines. Cell Stem Cell 33, 176–183. 10.1016/j.stem.2025.12.022 41564882

[B105] Iswandana R. Pham B. T. Suriguga S. Luangmonkong T. van Wijk L. A. Jansen Y. J. M. (2020). Murine precision-cut intestinal slices as a potential screening tool for antifibrotic drugs. Inflamm. Bowel Dis. 26, 678–686. 10.1093/ibd/izz329 31943022 PMC7150673

[B106] Jalan‐Sakrikar N. Brevini T. Huebert R. C. Sampaziotis F. (2023). Organoids and regenerative hepatology. Hepatology 77, 305–322. 10.1002/hep.32583 35596930 PMC9676408

[B107] Jennen D. G. J. Magkoufopoulou C. Ketelslegers H. B. van Herwijnen M. H. M. Kleinjans J. C. S. van Delft J. H. M. (2010). Comparison of HepG2 and HepaRG by whole-genome gene expression analysis for the purpose of chemical hazard identification. Toxicol. Sci. 115, 66–79. 10.1093/toxsci/kfq026 20106945

[B108] Jeong Y. E. Shea K. Ford K. A. (2025). Unraveling Caco-2 cells through functional and transcriptomic assessments. Regul. Toxicol. Pharmacol. 156, 105771. 10.1016/j.yrtph.2025.105771 39761805

[B109] Johnson A. C. Greenwood-Van Meerveld B. (2017). Critical evaluation of animal models of gastrointestinal disorders. Handb. Exp. Pharmacol. 239, 289–317. 10.1007/164_2016_120 28176046

[B110] Ju R. Tian S. Shang Y. Ma S. Zhang M. Liu J. (2024). Hepatocyte-like cells and liver organoids: the application of iPSCs and their derivants for treating liver diseases. Mater. Adv. 5, 8419–8431. 10.1039/d4ma00373j

[B111] Jung S. M. Kim S. (2021). *In vitro* models of the small intestine for studying intestinal diseases. Front. Microbiol. 12, 767038. 10.3389/fmicb.2021.767038 35058894 PMC8765704

[B112] Kadam O. Gumathannavar R. Basu K. Saini N. Sapre N. Wankar S. (2025). Applications of biopolymer scaffolds for intestinal delivery of drug-loaded nano-biomaterials: a review. Discov. Mater 5, 133. 10.1007/s43939-025-00321-8

[B113] Kaden T. Alonso-Román R. Stallhofer J. Gresnigt M. S. Hube B. Mosig A. S. (2025). Leveraging organ-on-chip models to investigate host–microbiota dynamics and targeted therapies for inflammatory bowel disease. Adv. Healthc. Mater. 14, 2402756. 10.1002/adhm.202402756 39491534 PMC12004439

[B114] Kang S. Park S. E. Huh D. D. (2021). Organ-on-a-chip technology for nanoparticle research. Nano Converg. 8, 20. 10.1186/s40580-021-00270-x 34236537 PMC8266951

[B115] Kararli T. T. (1995). Comparison of the gastrointestinal anatomy, physiology, and biochemistry of humans and commonly used laboratory animals. Biopharm. Drug Dispos. 16, 351–380. 10.1002/bdd.2510160502 8527686

[B116] Kasendra M. Tovaglieri A. Sontheimer-Phelps A. Jalili-Firoozinezhad S. Bein A. Chalkiadaki A. (2018). Development of a primary human small Intestine-on-a-Chip using biopsy-derived organoids. Sci. Rep-Uk 8, 2871. 10.1038/s41598-018-21201-7 29440725 PMC5811607

[B117] Kasendra M. Luc R. Yin J. Manatakis D. V. Kulkarni G. Lucchesi C. (2020). Duodenum intestine-chip for preclinical drug assessment in a human relevant model. Elife 9. 10.7554/eLife.50135 31933478 PMC6959988

[B118] Kawase A. Takashima O. Tanaka S. Shimada H. Iwaki M. (2022). Diclofenac-induced cytotoxicity in direct and indirect Co-Culture of HepG2 cells with differentiated THP-1 cells. Int. J. Mol. Sci. 23, 8660. 10.3390/ijms23158660 35955793 PMC9368861

[B119] Kazgan N. Metukuri M. R. Purushotham A. Lu J. Rao A. Lee S. (2014). Intestine-specific deletion of SIRT1 in mice impairs DCoH2-HNF-1α-FXR signaling and alters systemic bile acid homeostasis. Gastroenterology 146, 1006–1016. 10.1053/j.gastro.2013.12.029 24389307 PMC4142427

[B120] Kiesler P. Fuss I. J. Strober W. (2015). Experimental models of inflammatory bowel diseases. Cell. Mol. Gastroenterol. Hepatol. 1, 154–170. 10.1016/j.jcmgh.2015.01.006 26000334 PMC4435576

[B121] Kim H. J. Huh D. Hamilton G. Ingber D. E. (2012). Human gut-on-a-chip inhabited by microbial flora that experiences intestinal peristalsis-like motions and flow. Lab a Chip 12, 2165–2174. 10.1039/c2lc40074j 22434367

[B122] Kim H. Y. Lee W. Liu X. Jang H. Sakane S. Carvalho-Gontijo Weber R. (2024). Protocol to generate human liver spheroids to study liver fibrosis induced by metabolic stress. Star. Protoc. 5, 103111. 10.1016/j.xpro.2024.103111 38833372 PMC11179098

[B123] Kim Y. Kim H. Kim Y. (2025a). Advancing hepatotoxicity assessment: current advances and future directions. Toxicol. Res. 41, 303–323. 10.1007/s43188-025-00289-w 40612519 PMC12214119

[B124] Kim Y. Kang M. Mamo M. G. Adisasmita M. Huch M. Choi D. (2025b). Liver organoids: current advances and future applications for hepatology. Clin. Mol. Hepatol. 31, S327–S348. 10.3350/cmh.2024.1040 39722609 PMC11925438

[B125] Klein S. Mueller D. Schevchenko V. Noor F. (2014). Long‐term maintenance of HepaRG cells in serum‐free conditions and application in a repeated dose study. J Appl. Toxicol. 34, 1078–1086. 10.1002/jat.2929 24114766

[B126] Kodzhahinchev V. Biancolin A. Bucking C. (2018). Quantification of Mg2+, Ca2+ and H+ transport by the gastrointestinal tract of the goldfish, *Carassius auratus*, using the scanning Ion-selective electrode technique (SIET). PLoS One 13, e0207782. 10.1371/journal.pone.0207782 30513099 PMC6279021

[B127] Kraski A. Migdał P. Klopfleisch R. Räckel C. Sharbati J. Heimesaat M. M. (2024). Structured multicellular intestinal spheroids (SMIS) as a standardized model for infection biology. Gut Pathog. 16, 47. 10.1186/s13099-024-00644-6 39289703 PMC11406839

[B128] Krause P. Saghatolislam F. Koenig S. Unthan-Fechner K. Probst I. (2009). Maintaining hepatocyte differentiation *in vitro* through co-culture with hepatic stellate cells. Vitro Cell.Dev.Biol.-Animal 45, 205–212. 10.1007/s11626-008-9166-1 19184253

[B129] Kruepunga N. Hakvoort T. B. M. Hikspoors J. P. J. M. Köhler S. E. Lamers W. H. (2019). Anatomy of rodent and human livers: what are the differences? Biochimica Biophysica Acta (BBA) - Mol. Basis Dis. 1865, 869–878. 10.1016/j.bbadis.2018.05.019 29842921

[B130] Krumdieck C. L. dos Santos J. Ho K.-J. (1980). A new instrument for the rapid preparation of tissue slices. Anal. Biochem. 104, 118–123. 10.1016/0003-2697(80)90284-5 6770714

[B131] Kunz-Schughart L. A. Freyer J. P. Hofstaedter F. Ebner R. (2004). The use of 3-D cultures for high-throughput screening: the multicellular spheroid model. SLAS Discov. 9, 273–285. 10.1177/1087057104265040 15191644

[B132] Kus M. Ibragimow I. Piotrowska-Kempisty H. (2023). Caco-2 cell line standardization with pharmaceutical requirements and *in vitro* model suitability for permeability assays. Pharmaceutics 15, 2523. 10.3390/pharmaceutics15112523 38004503 PMC10674574

[B133] Lai Y. H. D’Souza M. J. (2008). Microparticle transport in the human intestinal M cell model. J. Drug Target 16, 36–42. 10.1080/10611860701639848 18172818

[B134] Lancaster M. A. Knoblich J. A. (2014). Organogenesis in a dish: modeling development and disease using organoid technologies. Science 345, 1247125. 10.1126/science.1247125 25035496

[B135] Larsen E. H. (2002). Hans H. Ussing—Scientific work: contemporary significance and perspectives. Biochimica Biophysica Acta (BBA) - Biomembr. 1566, 2–15. 10.1016/s0005-2736(02)00592-8 12421533

[B136] Lautenschläger C. Schmidt C. Lehr C.-M. Fischer D. Stallmach A. (2013). PEG-Functionalized microparticles selectively target inflamed mucosa in inflammatory bowel disease. Eur. J. Pharm. Biopharm. 85, 578–586. 10.1016/j.ejpb.2013.09.016 24084650

[B137] Lee S. Y. Sung J. H. (2018). Gut–liver on a chip toward an *in vitro* model of hepatic steatosis. Biotechnol. Bioeng. 115, 2817–2827. 10.1002/bit.26793 29981260

[B138] Lehmann R. Lee C. M. Shugart E. C. Benedetti M. Charo R. A. Gartner Z. (2019). Human organoids: a new dimension in cell biology. MBoC 30, 1129–1137. 10.1091/mbc.E19-03-0135 31034354 PMC6724519

[B139] Lerche-Langrand C. Toutain H. J. (2000). Precision-cut liver slices: characteristics and use for *in vitro* pharmaco-toxicology. Toxicology 153, 221–253. 10.1016/s0300-483x(00)00316-4 11090959

[B140] Li H. Sheppard D. N. Hug M. J. (2004). Transepithelial electrical measurements with the ussing chamber. J. Cyst. Fibros. 3, 123–126. 10.1016/j.jcf.2004.05.026 15463943

[B141] Li M. Vokral I. Evers B. de Graaf I. A. M. de Jager M. H. Groothuis G. M. M. (2018). Human and rat precision-cut intestinal slices as *ex vivo* models to study bile acid uptake by the apical sodium-dependent bile acid transporter. Eur. J. Pharm. Sci. 121, 65–73. 10.1016/j.ejps.2018.05.005 29751102

[B142] Li Y. Yang N. Chen J. Huang X. Zhang N. Yang S. (2020). Next-generation porcine intestinal organoids: an apical-out organoid model for swine enteric virus infection and immune response investigations. J. Virol. 94. 10.1128/JVI.01006-20 32796075 PMC7565635

[B143] Li Z. Yue M. Liu Y. Zhang P. Qing J. Liu H. (2022). Advances of engineered hydrogel organoids within the stem cell field: a systematic review. Gels 8, 379. 10.3390/gels8060379 35735722 PMC9222364

[B144] Liévin-Le Moal V. Servin A. L. (2013). Pathogenesis of human enterovirulent bacteria: lessons from cultured, fully differentiated human Colon cancer cell lines. Microbiol. Mol. Biol. Rev. 77, 380–439. 10.1128/MMBR.00064-12 24006470 PMC3811612

[B145] Lin F. Li X. Sun S. Li Z. Lv C. Bai J. (2023). Mechanically enhanced biogenesis of gut spheroids with instability-driven morphomechanics. Nat. Commun. 14, 6016. 10.1038/s41467-023-41760-2 37758697 PMC10533890

[B146] Liu Q. Zeng A. Liu Z. Wu C. Song L. (2022). Liver organoids: from fabrication to application in liver diseases. Front. Physiol. 13, 956244. 10.3389/fphys.2022.956244 35923228 PMC9340459

[B147] Loewa A. Feng J. J. Hedtrich S. (2023). Human disease models in drug development. Nat. Rev. Bioeng. 1, 545–559. 10.1038/s44222-023-00063-3 37359774 PMC10173243

[B148] López-Terrada D. Cheung S. W. Finegold M. J. Knowles B. B. (2009). Hep G2 is a hepatoblastoma-derived cell line. Hum. Pathol. 40, 1512–1515. 10.1016/j.humpath.2009.07.003 19751877

[B149] Lozoya-Agullo I. Araújo F. González-Álvarez I. Merino-Sanjuán M. González-Álvarez M. Bermejo M. (2017). Usefulness of Caco-2/HT29-MTX and Caco-2/HT29-MTX/Raji B coculture models to predict intestinal and colonic permeability compared to Caco-2 monoculture. Mol. Pharm. 14, 1264–1270. 10.1021/acs.molpharmaceut.6b01165 28263609

[B150] Lucchetti M. Aina K. O. Grandmougin L. Jäger C. Pérez Escriva P. Letellier E. (2024). An organ-on-chip platform for simulating drug metabolism along the gut-liver axis. Adv. Healthc. Mater. 13, e2303943. 10.1002/adhm.202303943 38452399

[B151] Lui C. Y. Amidon G. L. Berardi R. R. Fleisher D. Youngberg C. Dressman J. B. (1986). Comparison of gastrointestinal pH in dogs and humans: implications on the use of the beagle dog as a model for oral absorption in humans. J. Pharm. Sci. 75, 271–274. 10.1002/jps.2600750313 3701609

[B152] Lundquist P. Artursson P. (2016). Oral absorption of peptides and nanoparticles across the human intestine: opportunities, limitations and studies in human tissues. Adv. Drug Deliv. Rev. 106, 256–276. 10.1016/j.addr.2016.07.007 27496705

[B153] Lunney J. K. Van Goor A. Walker K. E. Hailstock T. Franklin J. Dai C. (2021). Importance of the pig as a human biomedical model. Sci. Transl. Med. 13, eabd5758. 10.1126/scitranslmed.abd5758 34818055

[B154] Majorova D. Atkins E. Martineau H. Vokral I. Oosterhuis D. Olinga P. (2021). Use of precision-cut tissue slices as a translational model to study host-pathogen interaction. Front. Vet. Sci. 8, 686088. 10.3389/fvets.2021.686088 34150901 PMC8212980

[B155] Malaguarnera G. Graute M. Homs Corbera A. (2021). The translational roadmap of the gut models, focusing on gut-on-chip. Open Res. Eur. 1, 62. 10.12688/openreseurope.13709.2 37645178 PMC10445823

[B156] Martinec O. Biel C. de Graaf I. A. M. Huliciak M. de Jong K. P. Staud F. (2021). Rifampicin induces gene, protein, and activity of P-Glycoprotein (ABCB1) in human precision-cut intestinal slices. Front. Pharmacol. 12, 684156. 10.3389/fphar.2021.684156 34177592 PMC8220149

[B157] Martini E. Krug S. M. Siegmund B. Neurath M. F. Becker C. (2017). Mend your fences: the epithelial barrier and its relationship with mucosal immunity in inflammatory bowel disease. Cell. Mol. Gastroenterology Hepatology 4, 33–46. 10.1016/j.jcmgh.2017.03.007 28560287 PMC5439240

[B158] Maurer M. Gresnigt M. S. Last A. Wollny T. Berlinghof F. Pospich R. (2019). A three-dimensional immunocompetent intestine-on-chip model as *in vitro* platform for functional and microbial interaction studies. Biomaterials 220, 119396. 10.1016/j.biomaterials.2019.119396 31398556

[B159] Mazza G. Telese A. Al-Akkad W. Frenguelli L. Levi A. Marrali M. (2019). Cirrhotic human liver extracellular matrix 3D scaffolds promote smad-dependent TGF-β1 epithelial mesenchymal transition. Cells 9, 83. 10.3390/cells9010083 31905709 PMC7017194

[B160] McNamara B. Winter D. C. Cuffe J. E. O’Sullivan G. C. Harvey B. J. (1999). Basolateral K^+^ channel involvement in forskolin‐activated chloride secretion in human Colon. J. Physiology 519, 251–260. 10.1111/j.1469-7793.1999.0251o.x 10432355 PMC2269479

[B161] Mendes C. Meirelles G. C. Silva M. A. S. Ponchel G. (2018). Intestinal permeability determinants of norfloxacin in ussing chamber model. Eur. J. Pharm. Sci. 121, 236–242. 10.1016/j.ejps.2018.05.030 29860116

[B162] Meroni M. Paolini E. Longo M. Piciotti R. Tria G. Fargion S. (2022). Recreating gut-liver axis during NAFLD onset by using a Caco-2/HepG2 co-culture system. Metabolism and target organ damage. 10.20517/mtod.2021.19

[B163] Messelmani T. Morisseau L. Sakai Y. Legallais C. Le Goff A. Leclerc E. (2022). Liver organ-on-chip models for toxicity studies and risk assessment. Lab. Chip 22, 2423–2450. 10.1039/d2lc00307d 35694831

[B164] Michiba K. Maeda K. Shimomura O. Miyazaki Y. Hashimoto S. Oda T. (2022). Usefulness of human jejunal spheroid–derived differentiated intestinal epithelial cells for the prediction of intestinal drug absorption in humans. Drug Metabolism Dispos. 50, 204–213. 10.1124/dmd.121.000796 34992074

[B165] Michiba K. Namai M. Hashimoto Y. Shimomura O. Miyazaki Y. Hashimoto S. (2025). Characterization of intestinal transporters in human ileal spheroid–derived differentiated cells for the prediction of intestinal drug absorption. Drug Metabolism Dispos. 53, 100075. 10.1016/j.dmd.2025.100075 40319556

[B166] Mitrofanova O. Nikolaev M. Xu Q. Broguiere N. Cubela I. Camp J. G. (2024). Bioengineered human Colon organoids with in vivo-like cellular complexity and function. Cell Stem Cell 31, 1175–1186.e7. 10.1016/j.stem.2024.05.007 38876106

[B167] Morelli M. Kurek D. Ng C. P. Queiroz K. (2023). Gut-on-a-Chip models: current and future perspectives for host-microbial interactions research. Biomedicines 11, 619. 10.3390/biomedicines11020619 36831155 PMC9953162

[B280] Morisseau L. (2023). Development of a human non-alcoholic fatty liver disease (NAFLD) model using organ-on-chip technology. Compiègne, France: Université de Technologie de Compiègne.

[B168] Morisseau L. (2026). Development of a human non-alcoholic fatty liver disease (NAFLD) model using organ-on-chip technology.

[B169] Nasser Y. Boeckxstaens G. E. Wouters M. M. Schemann M. Vanner S. (2014). Using human intestinal biopsies to study the pathogenesis of irritable bowel syndrome. Neurogastroenterol. Motil. 26, 455–469. 10.1111/nmo.12316 24602069

[B170] Natoli M. Leoni B. D. D’Agnano I. Zucco F. Felsani A. (2012). Good Caco-2 cell culture practices. Toxicol. Vitro 26, 1243–1246. 10.1016/j.tiv.2012.03.009 22465559

[B171] Nguyen O. T. P. Misun P. M. Hierlemann A. Lohasz C. (2024). A versatile intestine-on-chip system for deciphering the immunopathogenesis of inflammatory bowel disease. Adv. Healthc. Mater. 13, e2302454. 10.1002/adhm.202302454 38253407 PMC11468350

[B173] Nikolaev M. Mitrofanova O. Broguiere N. Geraldo S. Dutta D. Tabata Y. (2020). Homeostatic mini-intestines through scaffold-guided organoid morphogenesis. Nature 585, 574–578. 10.1038/s41586-020-2724-8 32939089

[B174] Niu X. de Graaf I. A. Groothuis G. M. (2013). Evaluation of the intestinal toxicity and transport of xenobiotics utilizing precision-cut slices. Xenobiotica 43, 73–83. 10.3109/00498254.2012.729870 23106567

[B175] Noel G. Baetz N. W. Staab J. F. Donowitz M. Kovbasnjuk O. Pasetti M. F. (2017). A primary human macrophage-enteroid co-culture model to investigate mucosal gut physiology and host-pathogen interactions. Sci. Rep-Uk 7, 45270. 10.1038/srep45270 28345602 PMC5366908

[B176] Noorman L. van der Hee B. Gilbert M. S. de Vries S. van der Hoek S. Gerrits W. J. J. (2024). Assessing seromuscular layer and serosa removal on intestinal permeability measurements in weaned piglet everted sac segments. J. Anim. Sci. 102, skae148. 10.1093/jas/skae148 38804653 PMC11222984

[B177] Nunes R. Silva C. Chaves L. (2016). Tissue-based *in vitro* and *ex vivo* models for intestinal permeability studies. in Concepts and models for drug permeability studies 203–236. (Elsevier) 10.1016/b978-0-08-100094-6.00013-4

[B178] Oami T. Coopersmith C. M. (2021). Measurement of intestinal permeability during sepsis. Methods Mol. Biol. 2321, 169–175. 10.1007/978-1-0716-1488-4_15 34048016 PMC8301743

[B179] Obeid D. A. Mir T. A. Alzhrani A. Altuhami A. Shamma T. Ahmed S. (2024). Using liver organoids as models to study the pathobiology of rare liver diseases. Biomedicines 12, 446. 10.3390/biomedicines12020446 38398048 PMC10887144

[B180] Obeid P. J. Yammine P. El-Nakat H. Kassab R. Tannous T. Nasr Z. (2024). Organ-On-a-Chip devices: technology progress and challenges. Chembiochem e202400580, e202400580. 10.1002/cbic.202400580 39183173

[B181] Odanga J. J. Gianulis E. Whaley L. LeCluyse E. L. Presnell S. Weaver J. R. (2023). An all-human hepatic culture system for drug development applications. JoVE 65992. 10.3791/65992 37930008

[B182] Olinga P. Schuppan D. (2013). Precision-cut liver slices: a tool to model the liver *ex vivo* . J. Hepatol. 58, 1252–1253. 10.1016/j.jhep.2013.01.009 23336979

[B183] Olson H. Betton G. Robinson D. Thomas K. Monro A. Kolaja G. (2000). Concordance of the toxicity of pharmaceuticals in humans and in animals. Regul. Toxicol. Pharmacol. 32, 56–67. 10.1006/rtph.2000.1399 11029269

[B184] Osonoi S. Takebe T. (2024). Organoid-guided precision hepatology for metabolic liver disease. J. Hepatology 80, 805–821. 10.1016/j.jhep.2024.01.002 38237864 PMC11828489

[B185] Othman A. Ehnert S. Dropmann A. Ruoß M. Nüssler A. K. Hammad S. (2020). Precision-cut liver slices as an alternative method for long-term hepatotoxicity studies. Archives Toxicol. 94, 2889–2891. 10.1007/s00204-020-02861-9 32683516

[B186] Özkan A. LoGrande N. T. Feitor J. F. Goyal G. Ingber D. E. (2024). Intestinal organ chips for disease modelling and personalized medicine. Nat. Rev. Gastro Hepat. 21, 751–773. 10.1038/s41575-024-00968-3 39192055

[B187] Padberg F. Höper T. Henkel S. Driesch D. Luch A. Zellmer S. (2021). Novel indirect co-culture of immortalised hepatocytes with monocyte derived macrophages is characterised by pro-inflammatory cytokine networks. Toxicol. Vitro 73, 105134. 10.1016/j.tiv.2021.105134 33662514

[B188] Palma E. Doornebal E. J. Chokshi S. (2019). Precision-cut liver slices: a versatile tool to advance liver research. Hepatol. Int. 13, 51–57. 10.1007/s12072-018-9913-7 30515676 PMC6513823

[B189] Parker K. K. Tan J. Chen C. S. Tung L. (2008). Myofibrillar architecture in engineered cardiac myocytes. Circulation Res. 103, 340–342. 10.1161/CIRCRESAHA.108.182469 18635822 PMC3910252

[B190] Parlament E. (2021).Plans and actions to accelerate a transition to innovation without the use of animals in research, regulatory testing and education. 2784 (RSP)

[B191] Pearce S. C. Coia H. G. Karl J. P. Pantoja-Feliciano I. G. Zachos N. C. Racicot K. (2018). Intestinal *in vitro* and *ex vivo* models to study host-microbiome interactions and acute stressors. Front. Physiol. 9, 1584. 10.3389/fphys.2018.01584 30483150 PMC6240795

[B192] Perrin-Cocon L. Vidalain P. O. Jacquemin C. Aublin-Gex A. Olmstead K. Panthu B. (2021). A hexokinase isoenzyme switch in human liver cancer cells promotes lipogenesis and enhances innate immunity. Commun. Biol. 4, 217. 10.1038/s42003-021-01749-3 33594203 PMC7886870

[B193] Pham B. T. van Haaften W. T. Oosterhuis D. Nieken J. de Graaf I. A. M. Olinga P. (2015). Precision-cut rat, mouse, and human intestinal slices as novel models for the early-onset of intestinal fibrosis. Physiol. Rep. 3. 10.14814/phy2.12323 25907784 PMC4425951

[B194] Polentarutti B. I. Peterson A. L. Sjöberg A. K. Anderberg E. K. Utter L. M. Ungell A. L. (1999). Evaluation of viability of excised rat intestinal segments in the ussing chamber: investigation of morphology, electrical parameters, and permeability characteristics. Pharm. Res. 16, 446–454. 10.1023/a:1018890106045 10213378

[B195] Polidoro M. A. Ferrari E. Marzorati S. Lleo A. Rasponi M. (2021). Experimental liver models: from cell culture techniques to microfluidic organs-on-chip. Liver Int. 41, 1744–1761. 10.1111/liv.14942 33966344

[B196] Raasch M. Rennert K. Jahn T. Peters S. Henkel T. Huber O. (2015). Microfluidically supported biochip design for culture of endothelial cell layers with improved perfusion conditions. Biofabrication 7, 015013. 10.1088/1758-5090/7/1/015013 25727374

[B197] Rahman S. Ghiboub M. Donkers J. M. van de Steeg E. van Tol E. A. F. Hakvoort T. B. M. (2021). The progress of intestinal epithelial models from cell lines to gut-on-chip. Int. J. Mol. Sci. 22, 13472. 10.3390/ijms222413472 34948271 PMC8709104

[B198] Rauth S. Karmakar S. Batra S. K. Ponnusamy M. P. (2021). Recent advances in organoid development and applications in disease modeling. Biochim. Biophys. Acta Rev. Cancer 1875, 188527. 10.1016/j.bbcan.2021.188527 33640383 PMC8068668

[B199] Rescigno M. (2015). Intestinal epithelial spheroids: new tools for studying gastrointestinal diseases. Gut 64, 859–860. 10.1136/gutjnl-2014-307809 25183204

[B200] Rezvani M. Vallier L. Guillot A. (2023). Modeling nonalcoholic fatty liver disease in the dish using human-specific platforms: strategies and limitations. Cell. Mol. Gastroenterology Hepatology 15, 1135–1145. 10.1016/j.jcmgh.2023.01.014 36740045 PMC10031472

[B201] Richter S. H. (2024). Challenging current scientific practice: how a shift in research methodology could reduce animal use. Lab. Anim. 53, 9–12. 10.1038/s41684-023-01308-9 38172390 PMC10766537

[B202] Rodrigues D. B. Failla M. L. (2021). Intestinal cell models for investigating the uptake, metabolism and absorption of dietary nutrients and bioactive compounds. Curr. Opin. Food Sci. 41, 169–179. 10.1016/j.cofs.2021.04.002

[B203] Roh T. T. Chen Y. Paul H. T. Guo C. Kaplan D. L. (2019). 3D bioengineered tissue model of the large intestine to study inflammatory bowel disease. Biomaterials 225, 119517. 10.1016/j.biomaterials.2019.119517 31580968 PMC6908826

[B204] Rose K. A. Holman N. S. Green A. M. Andersen M. E. LeCluyse E. L. (2016). Co-culture of hepatocytes and kupffer cells as an *in vitro* model of inflammation and drug-induced hepatotoxicity. J. Pharm. Sci. 105, 950–964. 10.1016/S0022-3549(15)00192-6 26869439 PMC5330391

[B205] Rosso F. Giordano A. Barbarisi M. Barbarisi A. (2004). From Cell–ECM interactions to tissue engineering. J. Cell. Physiol. 199, 174–180. 10.1002/jcp.10471 15039999

[B206] Ruan Y. Li X. You L. Chen J. Shen Y. Zhang J. (2021). Effect of pharmaceutical excipients on intestinal absorption of metformin *via* organic cation-selective transporters. Mol. Pharm. 18, 2198–2207. 10.1021/acs.molpharmaceut.0c01104 33956455

[B207] Ruoß M. Vosough M. Königsrainer A. Nadalin S. Wagner S. Sajadian S. (2020). Towards improved hepatocyte cultures: progress and limitations. Food Chem. Toxicol. 138, 111188. 10.1016/j.fct.2020.111188 32045649

[B208] Samy K. E. Levy E. S. Phong K. Demaree B. Abate A. R. Desai T. A. (2019). Human intestinal spheroids cultured using sacrificial micromolding as a model system for studying drug transport. Sci. Rep. 9, 9936. 10.1038/s41598-019-46408-0 31289365 PMC6616551

[B209] Sant S. Johnston P. A. (2017). The production of 3D tumor spheroids for cancer drug discovery. Technol. 23, 27–36. 10.1016/j.ddtec.2017.03.002 28647083 PMC5497458

[B210] Scharl M. Frei S. Pesch T. Kellermeier S. Arikkat J. Frei P. (2013). Interleukin-13 and transforming growth factor β synergise in the pathogenesis of human intestinal fistulae. Gut 62, 63–72. 10.1136/gutjnl-2011-300498 22287592

[B211] Schicht G. Seidemann L. Haensel R. Seehofer D. Damm G. (2022). Critical investigation of the usability of hepatoma cell lines HepG2 and Huh7 as models for the metabolic representation of resectable hepatocellular carcinoma. Cancers 14, 4227. 10.3390/cancers14174227 36077764 PMC9454736

[B212] Schmidt C. Lautenschlaeger C. Collnot E. M. Schumann M. Bojarski C. Schulzke J. D. (2013). Nano- and microscaled particles for drug targeting to inflamed intestinal mucosa—A first *in vivo* study in human patients. J. Control. Release 165, 139–145. 10.1016/j.jconrel.2012.10.019 23127508

[B213] Senoo H. Kojima N. Sato M. (2007). Vitamin A-storing cells (stellate cells). Vitam. Horm. 75, 131–159. 10.1016/S0083-6729(06)75006-3 17368315

[B214] Shah P. Fritz J. V. Glaab E. Desai M. S. Greenhalgh K. Frachet A. (2016). A microfluidics-based *in vitro* model of the gastrointestinal human-microbe interface. Nat. Commun. 7, 11535. 10.1038/ncomms11535 27168102 PMC4865890

[B215] Shin W. Hinojosa C. D. Ingber D. E. Kim H. J. (2019). Human intestinal morphogenesis controlled by transepithelial morphogen gradient and flow-dependent physical cues in a microengineered Gut-on-a-Chip. iScience 15, 391–406. 10.1016/j.isci.2019.04.037 31108394 PMC6526295

[B216] Siwczak F. Loffet E. Kaminska M. Koceva H. Mahe M. M. Mosig A. S. (2021). Intestinal stem cell-on-chip to study human host-microbiota interaction. Front. Immunol. 12, 798552. 10.3389/fimmu.2021.798552 34938299 PMC8685395

[B217] Sjögren E. Abrahamsson B. Augustijns P. Becker D. Bolger M. B. Brewster M. (2014). *In vivo* methods for drug absorption – comparative physiologies, model selection, correlations with *in vitro* methods (IVIVC), and applications for formulation/API/excipient characterization including food effects. Eur. J. Pharm. Sci. 57, 99–151. 10.1016/j.ejps.2014.02.010 24637348

[B218] Sjögren E. Eriksson J. Vedin C. Breitholtz K. Hilgendorf C. (2016). Excised segments of rat small intestine in ussing chamber studies: a comparison of native and stripped tissue viability and permeability to drugs. Int. J. Pharm. 505, 361–368. 10.1016/j.ijpharm.2016.03.063 27073083

[B219] Snelson M. Vanuytsel T. Marques F. Z. (2024). Breaking the barrier: the role of gut epithelial permeability in the pathogenesis of hypertension. Curr. Hypertens. Rep. 26, 369–380. 10.1007/s11906-024-01307-2 38662328 PMC11324679

[B220] Sontheimer-Phelps A. Chou D. B. Tovaglieri A. Ferrante T. C. Duckworth T. Fadel C. (2020). Human Colon-on-a-Chip enables continuous *in vitro* analysis of Colon mucus layer accumulation and physiology. Cell. Mol. Gastroenterology Hepatology 9, 507–526. 10.1016/j.jcmgh.2019.11.008 31778828 PMC7036549

[B221] Soto A. Spongberg C. Martinino A. Giovinazzo F. (2024). Exploring the multifaceted landscape of MASLD: a comprehensive synthesis of recent studies, from pathophysiology to organoids and beyond. Biomedicines 12, 397. 10.3390/biomedicines12020397 38397999 PMC10886580

[B222] Spence J. R. Mayhew C. N. Rankin S. A. Kuhar M. F. Vallance J. E. Tolle K. (2011). Directed differentiation of human pluripotent stem cells into intestinal tissue *in vitro* . Nature 470, 105–109. 10.1038/nature09691 21151107 PMC3033971

[B223] Srinivasan B. Kolli A. R. Esch M. B. Abaci H. E. Shuler M. L. Hickman J. J. (2015). TEER measurement techniques for *in vitro* barrier model systems. J. Lab. Autom. 20, 107–126. 10.1177/2211068214561025 25586998 PMC4652793

[B224] Steffansen B. Pedersen M. D. L. Laghmoch A. M. Nielsen C. U. (2017). SGLT1-Mediated transport in Caco-2 cells is highly dependent on cell bank origin. J. Pharm. Sci. 106, 2664–2670. 10.1016/j.xphs.2017.04.033 28454747

[B225] Stockmann M. Gitter A. H. Sorgenfrei D. Fromm M. Schulzke J. D. (1999). Low edge damage container insert that adjusts intestinal forceps biopsies into ussing chamber systems. Pflugers Arch. 438, 107–112. 10.1007/s004240050886 10370094

[B226] Streekstra E. J. Kiss M. van den Heuvel J. Nicolaï J. van den Broek P. Botden S. M. B. I. (2022). A proof of concept using the ussing chamber methodology to study pediatric intestinal drug transport and age-dependent differences in absorption. Clin. Transl. Sci. 15, 2392–2402. 10.1111/cts.13368 35962572 PMC9579398

[B227] Sui M. Liu H. Ren Y. Liu D. (2011). Study on Zn relative concentration and state in sheep duodenum by XAFS. Biol. Trace Elem. Res. 143, 240–250. 10.1007/s12011-010-8843-6 20848237

[B228] Taelman J. Diaz M. Guiu J. (2022). Human intestinal organoids: promise and challenge. Front. Cell Dev. Biol. 10, 854740. 10.3389/fcell.2022.854740 35359445 PMC8962662

[B229] Takebe T. Sekine K. Suzuki Y. Enomura M. Tanaka S. Ueno Y. (2012). Self-organization of human hepatic organoid by recapitulating organogenesis *in vitro* . Transplant. Proc. 44, 1018–1020. 10.1016/j.transproceed.2012.02.007 22564614

[B230] Takebe T. Imai R. Ono S. (2018). The current status of drug discovery and development as originated in United States academia: the influence of industrial and academic collaboration on drug discovery and development. Clin. Transl. Sci. 11, 597–606. 10.1111/cts.12577 29940695 PMC6226120

[B231] Takemura A. Gong S. Sato T. Kawaguchi M. Sekine S. Kazuki Y. (2021). Evaluation of Parent- and metabolite-induced mitochondrial toxicities using CYP-introduced HepG2 cells. J. Pharm. Sci. 110, 3306–3312. 10.1016/j.xphs.2021.06.001 34097978

[B232] Takenaka T. Harada N. Kuze J. Chiba M. Iwao T. Matsunaga T. (2016). Application of a human intestinal epithelial cell monolayer to the prediction of oral drug absorption in humans as a superior alternative to the Caco-2 cell monolayer. J. Pharm. Sci. 105, 915–924. 10.1016/j.xphs.2015.11.035 26869436

[B233] Tevlek A. Kecili S. Ozcelik O. S. Kulah H. Tekin H. C. (2023). Spheroid engineering in microfluidic devices. ACS Omega 8, 3630–3649. 10.1021/acsomega.2c06052 36743071 PMC9893254

[B234] Thomas D. P. Zhang J. Nguyen N.-T. Ta H. T. (2023). Microfluidic Gut-on-a-Chip: fundamentals and challenges. Biosensors 13, 136. 10.3390/bios13010136 36671971 PMC9856111

[B235] Thomson A. Smart K. Somerville M. S. Lauder S. N. Appanna G. Horwood J. (2019). The ussing chamber system for measuring intestinal permeability in health and disease. BMC Gastroenterol. 19, 98. 10.1186/s12876-019-1002-4 31221083 PMC6585111

[B236] Tian C. Yang M. F. Xu H. M. Zhu M. Z. Yue N. N. Zhang Y. (2023). Stem cell-derived intestinal organoids: a novel modality for IBD. Cell Death Discov. 9, 255. 10.1038/s41420-023-01556-1 37479716 PMC10362068

[B237] Trietsch S. J. Naumovska E. Kurek D. Setyawati M. C. Vormann M. K. Wilschut K. J. (2017). Membrane-free culture and real-time barrier integrity assessment of perfused intestinal epithelium tubes. Nat. Commun. 8, 262. 10.1038/s41467-017-00259-3 28811479 PMC5557798

[B238] Tselekouni P. Mohseni-Garakani M. Papa S. Kim S. Y. Avramoglu R. K. Wertheimer M. R. (2026). A 3D alginate-gelatin Co-Culture model to study epithelial-stromal interactions in the gut. Gels 12, 70. 10.3390/gels12010070 41590095 PMC12841391

[B172] United States. Department of Health and Human Services, issuing body (2017). Waiver of in vivo bioavailability and bioequivalence studies for immediate-release solid oral dosage forms based on a biopharmaceutics classification system guidance for industry. Center for Drug Evaluation and Research. Available online at: http://resource.nlm.nih.gov/101720038.

[B239] VanDussen K. L. Marinshaw J. M. Shaikh N. Miyoshi H. Moon C. Tarr P. I. (2015). Development of an enhanced human gastrointestinal epithelial culture system to facilitate patient-based assays. Gut 64, 911–920. 10.1136/gutjnl-2013-306651 25007816 PMC4305344

[B240] Vanhoeijen R. Okkelman I. A. Rogier N. Sedlačík T. Stöbener D. D. Devriendt B. (2025). Poly(2-alkyl-2-oxazoline) hydrogels as synthetic matrices for multicellular spheroid and intestinal organoid cultures. Biomacromolecules 26, 1860–1872. 10.1021/acs.biomac.4c01627 39898884

[B241] Velliou R.-I. Giannousi E. Ralliou C. Kassi E. Chatzigeorgiou A. (2024). *Ex Vivo* tools and models in MASLD research. Cells 13, 1827. 10.3390/cells13221827 39594577 PMC11592755

[B242] Vergauwen H. (2015). “The IPEC-J2 cell line,” in The impact of food bioactives on health. Editor Verhoeckx K. (Cham: Springer International Publishing), 125–134. 10.1007/978-3-319-16104-4_12

[B243] Viswanathan V. K. Weflen A. Koutsouris A. Roxas J. L. Hecht G. (2008). Enteropathogenic*E. Coli*-induced barrier function alteration is not a consequence of host cell apoptosis. Am. J. Physiol. Gastrointest. Liver Physiol. 294, G1165–G1170. 10.1152/ajpgi.00596.2007 18356531 PMC3327053

[B244] Wang L. Ding L. Du Z. Yu Z. Liu J. (2019). Hydrolysis and transport of egg white-derived peptides in Caco-2 cell monolayers and everted rat sacs. J. Agric. Food Chem. 67, 4839–4848. 10.1021/acs.jafc.9b01904 30969123

[B245] Wang L. Han J. Su W. Li A. Zhang W. Li H. (2023). Gut-on-a-chip for exploring the transport mechanism of Hg(II). Microsyst. Nanoeng. 9, 2. 10.1038/s41378-022-00447-2 36597512 PMC9805456

[B246] Ware B. R. Khetani S. R. (2017). Engineered liver platforms for different phases of drug development. Trends Biotechnol. 35, 172–183. 10.1016/j.tibtech.2016.08.001 27592803 PMC5253249

[B247] Watson C. L. Mahe M. M. Múnera J. Howell J. C. Sundaram N. Poling H. M. (2014). An *in vivo* model of human small intestine using pluripotent stem cells. Nat. Med. 20, 1310–1314. 10.1038/nm.3737 25326803 PMC4408376

[B248] Weiskirchen R. (2022). Established liver cell lines: are you sure to have the right ones? Livers 2, 171–177. 10.3390/livers2030015

[B16] Westerhout J. Wortelboer H. Verhoeckx K. (2015). “Ussing chamber in COST Action FA1005,” in The impact of food bio-actives on gut health: in vitro and ex vivo models”. Cham: Springer International Publishing, 263–273. 10.1007/978-3-319-16104-4_24

[B249] Westra I. M. Mutsaers H. A. M. Luangmonkong T. Hadi M. Oosterhuis D. de Jong K. P. (2016). Human precision-cut liver slices as a model to test antifibrotic drugs in the early onset of liver fibrosis. Toxicol Vitro 35, 77–85. 10.1016/j.tiv.2016.05.012 27235791

[B250] Wilson T. H. Wiseman G. (1954). The use of sacs of everted small intestine for the study of the transference of substances from the mucosal to the serosal surface. J. Physiol. 123, 116–125. 10.1113/jphysiol.1954.sp005036 13131249 PMC1366157

[B251] Wu X. Roberto J. B. Knupp A. Kenerson H. L. Truong C. D. Yuen S. Y. (2018). Precision-cut human liver slice cultures as an immunological platform. J. Immunol. Methods 455, 71–79. 10.1016/j.jim.2018.01.012 29408707 PMC6689534

[B252] Xia M. Varmazyad M. Pla-Palacín I. Gavlock D. C. DeBiasio R. LaRocca G. (2024). Comparison of wild-type and high-risk PNPLA3 variants in a human biomimetic liver microphysiology system for metabolic dysfunction-associated steatotic liver disease precision therapy. Front. Cell Dev. Biol. 12, 1423936. 10.3389/fcell.2024.1423936 39324073 PMC11422722

[B253] Xu Y. Shrestha N. Préat V. Beloqui A. (2021). An overview of *in vitro,*, *ex vivo* and *in vivo* models for studying the transport of drugs across intestinal barriers. Adv. Drug Deliv. Rev. 175, 113795. 10.1016/j.addr.2021.05.005 33989702

[B254] Youhanna S. Kemas A. M. Wright S. C. Zhong Y. Klumpp B. Klein K. (2025). Chemogenomic screening in a patient-derived 3D fatty liver disease model reveals the CHRM1-TRPM8 axis as a novel module for targeted intervention. Adv. Sci. 12, 2407572. 10.1002/advs.202407572 39605182 PMC11744578

[B255] Zhang Z. Xu H. Mazza G. Zhang M. Frenguelli L. Liu Q. (2019). Decellularized human liver scaffold‐based three‐dimensional culture system facilitate hepatitis B virus infection. J. Biomed. Mater. Res. 107, 1744–1753. 10.1002/jbm.a.36690 30963688

[B256] Zhang Q. Nguyen A. L. Shi S. Hill C. Wilder-Smith P. Krasieva T. B. (2012). Three-dimensional spheroid culture of human gingiva-derived mesenchymal stem cells enhances mitigation of chemotherapy-induced oral mucositis. Stem Cells Dev. 21, 937–947. 10.1089/scd.2011.0252 21689066 PMC3315752

[B257] Zhang H. Qin Y. Jia M. Li L. Zhang W. Li L. (2023). A gastric cancer patient-derived three-dimensional cell spheroid culture model. Am. J. Cancer Res. 13, 964–975. 37034210 PMC10077029

[B258] Zhao Z. Chen X. Dowbaj A. M. Sljukic A. Bratlie K. Lin L. (2022). Organoids. Nat. Rev. Methods Prim. 2, 94. 10.1038/s43586-022-00174-y 37325195 PMC10270325

[B259] Zhao Q. De Nardo W. Wang R. Zhong Y. Keles U. Zhao L. N. (2025). Omics-based insights into human liver reveal GTPase-driven mechanisms of MASLD progression in obesity. Preprint. 10.1101/2025.01.13.632747

[B260] Zhu L. Cheng C. Liu S. Yang L. Han P. Cui T. (2023). Advancements and application prospects of three-dimensional models for primary liver cancer: a comprehensive review. Front. Bioeng. Biotechnol. 11, 1343177. 10.3389/fbioe.2023.1343177 38188493 PMC10771299

[B261] Zweibaum A. Laburthe M. Grasset E. Louvard D. (1991). “Use of cultured cell lines in studies of intestinal cell differentiation and function,” in Comprehensive physiology. Editor Terjung R. (Wiley), 223–255. 10.1002/cphy.cp060407

